# Exploring Geometrical, Electronic and Spectroscopic Properties of 2-Nitroimidazole-Based Radiopharmaceuticals via Computational Chemistry Methods

**DOI:** 10.3390/molecules29071505

**Published:** 2024-03-28

**Authors:** George Crișan, Ștefan Stan, Vasile Chiș

**Affiliations:** 1 Faculty of Physics, Babeș-Bolyai University, Str. M. Kogălniceanu 1, RO-400084 Cluj-Napoca, Romania; george.crisan@scjucluj.ro (G.C.); stefan.stan@ubbcluj.ro (Ș.S.); 2 Department of Nuclear Medicine, County Clinical Hospital, Clinicilor 3-5, RO-400006 Cluj-Napoca, Romania; 3 Institute for Research, Development and Innovation in Applied Natural Sciences, Babeș-Bolyai University, Str. Fântânele 30, RO-400327 Cluj-Napoca, Romania

**Keywords:** 2-nitroimidazole radiopharmaceuticals, PET, tumor hypoxia, computational spectroscopy

## Abstract

Tumor hypoxia plays an important role in the clinical management and treatment planning of various cancers. The use of 2-nitroimidazole-based radiopharmaceuticals has been the most successful for positron emission tomography (PET) and single photon emission computed tomography (SPECT) imaging probes, offering noninvasive means to assess tumor hypoxia. In this study we performed detailed computational investigations of the most used compounds for PET imaging, focusing on those derived from 2-nitroimidazole: fluoromisonidazole (FMISO), fluoroazomycin arabinoside (FAZA), fluoroetanidazole (FETA), fluoroerythronitroimidazole (FETNIM) and 2-(2-nitroimidazol-1-yl)-*N*-(2,2,3,3,3-pentafluoropropyl)acetamide (EF5). Conformational analysis, structural parameters, vibrational IR and Raman properties (within both harmonic and anharmonic approximations), as well as the NMR shielding tensors and spin-spin coupling constants were obtained by density functional theory (DFT) calculations and then correlated with experimental findings, where available. Furthermore, time-dependent DFT computations reveal insight into the excited states of the compounds. Our results predict a significant change in the conformational landscape of most of the investigated compounds when transitioning from the gas phase to aqueous solution. According to computational data, the 2-nitroimidazole moiety determines to a large extent the spectroscopic properties of its derivatives. Due to the limited structural information available in the current literature for the investigated compounds, the findings presented herein deepen the current understanding of the electronic structures of these five radiopharmaceuticals.

## 1. Introduction

**Figure 1:**
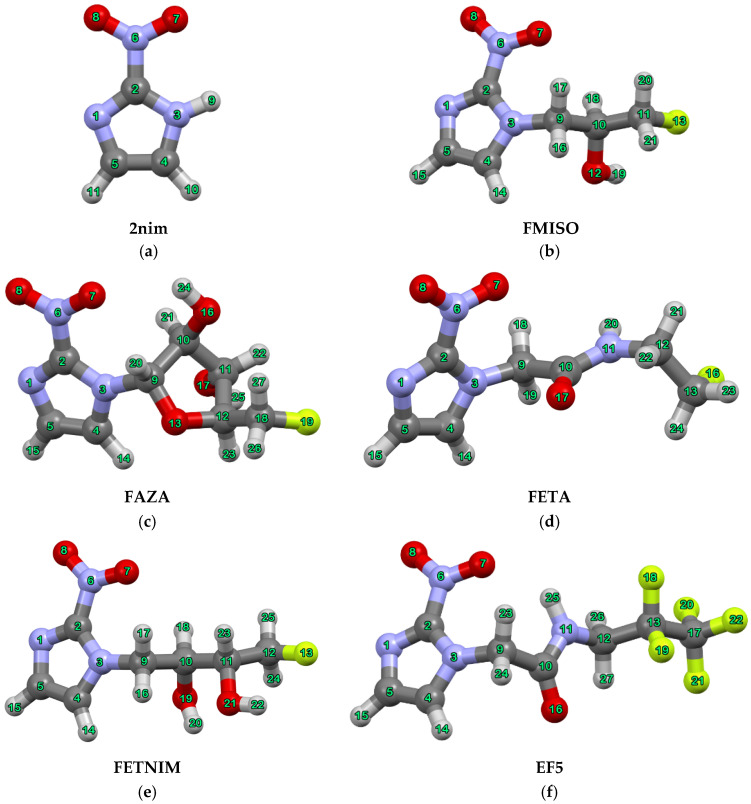
B3LYP/6-311+G(d,p) optimized structures of the investigated molecules, with atom numbering schemes (**a**) **2nim** (**b**) FMISO; (**c**) FAZA; (**d**) FETA; (**e**) FETNIM; (**f**) EF5. Atom color scheme: gray—C, light gray—H, light purple—N, red—O and light green—F.

Hypoxia is a common feature of the microenvironment of solid tumors and is associated with metastasis, recurrence and the development of resistance to radiotherapy, chemotherapy and immunotherapy [1,2,3]. There is a continuous effort to develop methods able to facilitate proper oncological treatment [2]. Among these methods, molecular imaging techniques like PET and SPECT, using nitroimidazole derivatives, have shown promising results [4,5]. Their effectiveness is due to their ability to undergo oxygen dependent reduction specifically in hypoxic cells. A significant number of ^18^F-labelled radiopharmaceuticals for hypoxia imaging have been successfully developed and evaluated in clinical trials [6]. Fluromisonidazole (FMISO) was the first 2-nitroimidazole (**2nim**) derivative to undergo clinical testing and remains the preferred standard in clinical imaging. However, its high lipophilicity results in low uptake and low-contrast images, thereby prompting the development of more optimized radiopharmaceuticals such as fluoroazomycin arabinoside (FAZA), fluroetanidazole (FETA), fluoroerythronitroimidazole (FETNIM) and 2-(2-nitroimidazol-1-yl)-*N*-(2,2,3,3,3-pentafluoropropyl) acetamide (EF5) (see Figure 1) [5].

The current understanding of the molecular mechanism of nitroimidazole compounds in hypoxia cells is limited. A recent study by Rashed et al. [7] investigated the mechanism of action of two nitroimidazoles (IAZA and FAZA) in a head and neck cancer model. They concluded that covalent binding of the investigated compounds to proteins can inhibit catalytic functions of critical cellular enzymes and they plan on further analyzing the proteins targeted by the nitroimidazoles. Once the targets are identified, studies of receptor–ligand binding could further advance the understanding of the mechanism of action and would require detailed structural (conformational) information of the nitroimidazole derivatives.

Thus, we consider that our results related to the structure and conformations of the investigated compounds could be useful for docking studies and ligand–receptor interactions [8,9]. The predicted spectroscopic properties might also be of help for drug monitoring [10], for drug detection or for pharmaceutical research and development [11].

Although extensively utilized in nuclear medicine, detailed information on their molecular and electronic configurations remains limited. Therefore, in this study, we present a computational structural analysis of the aforementioned radiopharmaceuticals. Despite their development beginning in the late 1980s, a review of the current literature reveals a lack of data regarding the electronic structures of these compounds. According to the Web of Science database, only 64 papers were found when searching for the keywords “radiopharmaceuticals” AND “DFT”.

Our aim was to provide a characterization of their conformational landscape and to determine the optimal structures of possible conformers, as well as to analyze their vibrational, NMR and electronic spectroscopic properties using density functional theory calculations. All these compounds show favorable properties for hypoxia imaging; however, there are still limitations in their implementation in the clinical setting [5]. Furthermore, compounds such as FMISO will act as a benchmark when evaluating the performance of new imaging agents. To this extent, we consider that a thorough structural characterization of these compounds could provide information that can be useful when optimizing and developing new agents.

## 2. Results and Discussion

### 2.1. Conformers and Boltzmann Population Analysis

The conformers of the five compounds in the gas phase were generated from the 2D relaxed potential energy surfaces (PES) defined by the dihedral angles shown in Table 1. In the case of FAZA, because of its low structural flexibility we investigated only the 1D PES generated by scanning the C4-N3-C9-C10 dihedral angle. The scanned dihedrals were incremented 24 times in steps of 15° over the 360° interval.

We identified 9 local minima for FMISO, 11 for FETNIM, 3 for FAZA and 2 for FETA and EF5. The geometries of the minima for each compound were further fully optimized at the B3LYP/6-311+G(d,p) level of theory, both in gas phase and water. These new geometry optimizations revealed a lower number of unique conformations for FMISO and FETNIM (5 and 7, respectively). The relative Boltzmann populations were calculated using the following formula:

(1) $$P_{i}=\frac{e^{-\frac{∆G_{i}}{k_{B}T}}}{\sum_{i}e^{-\frac{∆G_{i}}{k_{B}T}}}$$

where ∆*G_i_* are the relative (Gibbs) free energies, *k_B_* is the Boltzmann constant, and *T* is the temperature (*T* = 298 K) [12,13,14]. The Gibbs free energy was used for Boltzmann population calculations as recommended in ref. [12].

The structures of the most stable conformers in the gas phase and water are shown in Figure S1, while Boltzmann populations are presented in Table 2. Geometry re-optimizations in water show small changes in the structures of the conformers. On the other hand, as can be seen in Table 2, important changes are noted between the Boltzmann populations of the conformers when passing from the gas phase to the liquid state. Regarding the energetic order of the conformations for the five investigated compounds, the following matchings were observed:

FMISO: 1g ↔ 1w, 2g ↔ 3w, 3g ↔ 5w, 4g ↔ 2w, 5g ↔ 4w
FETNIM: 1g ↔ 1w, 2g ↔ 3w, 3g ↔ 2w, 4g ↔ 4w, 5g ↔ 5w, 6g ↔ 6w, 7g ↔ 7w
FAZA: 1g ↔ 1w, 2g ↔ 3w, 3g ↔ 2w
FETA: 1g ↔ 2w, 2g ↔ 1w
EF5: 1g ↔ 1w, 2g ↔ 2w

As an example, the above mentioned correspondences for FMISO means that the conformer 1g is the most stable in the gas phase. It exhibits structural similarity with the conformer 1w, which attains maximal stability in an aqueous environment. The 3g conformer, which ranks as the third most stable in the gas phase, experiences a decrease in relative stability in water, where it is ranked as the fifth most stable, denoted as 5w.

According to data collected in Table 2, in the case of FMISO, the most stable conformer in the gas phase remains the most stable in water as well, with a similar relative population 62.15% vs. 65.91%. The conformers 2g and 3g suffer a destabilization due to solvation and they transform into the 3w and 5w conformers in water, with the relative population of 7.59% and 2.33%, respectively. On the other hand, the conformer 4g shows an increase in relative population to 20.56% in water, becoming the second most stabilized.

For FETNIM, most conformers suffer a slight destabilization due to solvation effects; however, the conformer 3g shows an increase in relative population in water from 8.22% to 23.16%, becoming the second most stabilized in the aqueous environment.

Significant solvent destabilization effects can be observed for FAZA 1g and FETA 1g, while a large increase in relative population can be seen for the conformer FAZA 3g from 0.29% in gas phase to 28.46% in water. This change from the gas phase to water comes with a structural reconfiguration, mainly in the dihedral angles related to the oxolane group.

The two conformers of EF5 show negligible solvation effects and retain the same energetic order in the gas phase and water.

### 2.2. Vibrational Properties

#### 2.2.1. 2-Nitroimidazole

**Figure 2:**
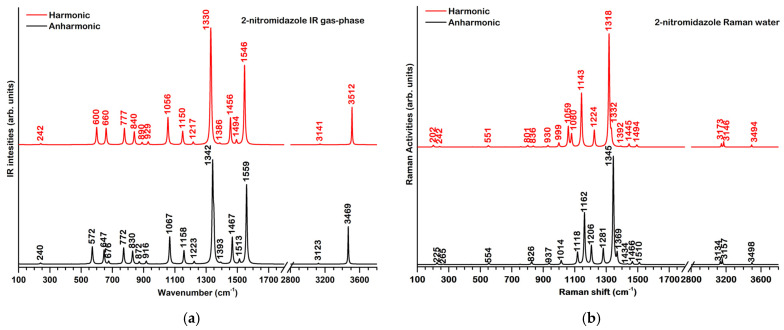
Theoretical IR spectrum in the gas phase (**a**) and Raman spectrum in water (**b**) for **2nim** in the harmonic (red) and anharmonic (black) approximation, at the B3LYP/6-311+G(d,p) level of theory.

The theoretical IR and Raman spectra of **2nim** in the harmonic (scaled frequencies) and anharmonic approximations in the gas phase and water are presented in Figure 2. The assignments of the most representative bands are given in Table 3.

In the fingerprint region of the IR spectrum, we note the most intense calculated bands at 1330 and 1546 cm^−1^, both involving the vibrations of the nitro group, assigned to the ν(C-N) + ν_sym_(NO_2_) and ν_asym_(NO_2_) normal modes. On the other hand, the most Raman active predicted mode (ν(C-N) + ν(C=C)) is calculated at 1318 and 1345 cm^−1^ by the harmonic and anharmonic approximations, respectively (see Figure 2b). This mode, together with those calculated at 1059/1206 and 1143/1162 cm^−1^ form the set of characteristic Raman bands for **2nim**.

At high wavenumbers, the three observed bands were assigned to a ν(NH) mode at 3512 cm^−1^ and two ν(CH) modes at 3165 and 3141 cm^−1^.

Experimental data for the IR spectrum of **2nim** were obtained from references [15,16]. As seen in Table 3, there is a good agreement between the experimental and computed IR wavenumbers. Expectedly, the experimental bands are generally better described by the anharmonic approximation with the exception of some observed bands at 623, 944, 3146 and 3164 cm^−1^ which are more closely reproduced by the scaled harmonic data.

#### 2.2.2. 2-Nitroimidazole Derivatives

**Figure 3:**
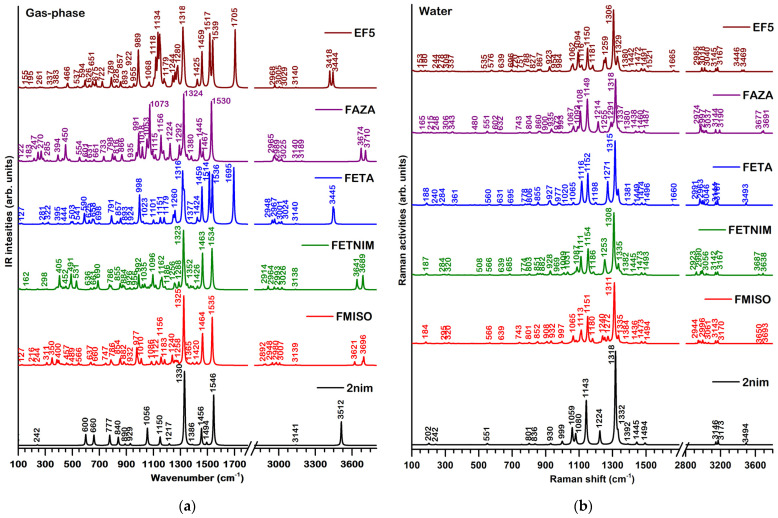
The harmonic (scaled) IR (gas-phase) (**a**) and Raman (water) (**b**) spectra for **2nim** and the five derivatives. The spectra for the derivatives are the Boltzmann-weighted averages of the most stable conformers.

The theoretical harmonic IR (gas-phase) and Raman (water) spectra of the five nitroimidazole derivatives are presented in Figure 3 (spectra of **2nim** are included for comparison purposes). The assignments of the most representative bands for each compound are available in the Supplementary Materials (Tables S1–S5).

We can distinctly observe characteristic spectral bands associated with **2nim** in the spectra of all five compounds. Such calculated bands include the ν(C-N)_imidazole_ + ν_sym_(NO_2_) mode at ~1330 cm^−1^, the ν(C-N)_imidazole_ + β(NH) + β(CH) mode at ~1456 cm^−1^, ν_asym_(NO_2_) at ~1546 cm^−1^ and two ν(CH) modes at ~ 3141 and 3165 cm^−1^. The specific wavenumbers for these bands can be found in Table 3. It is noteworthy that the first three bands exhibit a red-shift, whereas the second ν(CH) mode in FAZA experiences a blue-shift of 23 cm^−1^. Notably, the spectral bands at 997 and 1056 cm^−1^ do not have a corresponding mode in the calculations for the **2nim** derivatives.

The Boltzmann-weighted spectra presented in Figure 3 take into account the conformers with a Boltzmann population higher that 5%. Comparative figures illustrating the spectral variances among different conformers for each compound can be found in the Supplementary Materials (Figures S2–S6). We observe that the composite spectra are following the spectral landscape of the spectrum for the most stable conformer. For FMISO and FETNIM, significant differences in the spectral bands of the predicted conformers are observed, particularly in the lower fingerprint region. However, as the Boltzmann population for the most stable conformer is much larger, its contribution to the composite spectra outweighs that of the other conformers. A notable exception is evident in the composite spectra of FMISO where one can observe two distinct ν(OH) bands: at 3696 cm^−1^ originating from the conformers 1g and 2g, and at 3621 cm^−1^ attributed to the contribution of the conformer 3g (IR harmonic gas phase). In the case of FETA, both conformers exhibit similar vibrational patterns, without any obvious individual contributions. Finally, for EF5, where the Boltzmann populations of the two most stable conformers are very close, three doublets are discerned in the composite spectra which are due to the contributions of both conformers: 383 (1g)/371 (2g), 651 (1g)/675 (2g) and 3418 (1g)/3444 (2g) cm^−1^.

Conformer analysis and Boltzmann population analysis show an energetic reordering of the most stable conformers for each compound when considering water as a solvent. Radiopharmaceuticals are usually administered to patients through intravenous injections of aqueous solutions. Figure 3b presents the calculated harmonic Raman spectra for **2nim** and the Boltzmann-weighted averages of the most stable conformers for the five derivatives in water. In the case of FETNIM, FETA and FAZA, no specific conformer contributions can be observed due the similarities of the corresponding spectra for the conformers with the highest predicted populations. For EF5, one doublet can be discerned at 3469 (1w)/3446 (2w) and two conformer-specific bands at 203 (2w) and at 180 (1w) cm^−1^. In the case of FMISO, calculations predict significant conformer-specific characteristics in the averaged Raman spectrum weighted by the three most stable conformers in water: two doublets at 3693 (1w)/3650 (2w) and at 1272 (1w)/1253 (2w + 3w) cm^−1^, as well as the band at 1065 cm^−1^ arising due to the contributions of the conformers 1w and 3w and the band at 632 cm^−1^ due to the conformers 2w and 3w.

There are no significantly intense overtones or combination modes calculated for the anharmonic spectra. Figures presenting the comparison between the IR and Raman harmonic and anharmonic spectra of the most stable conformer in the gas phase are available in the Supplementary Materials for all compounds (Figures S7–S11). Calculated anharmonic wavenumbers are shown in the assignment tables in the Supplementary Materials Tables S1–S5.

In Table S1, we provide a tentative comparison with experimental IR data from Ref. [17] for FMISO. It is important to note that the reference only provides IR wavenumbers, without IR intensity. Therefore, our assignment in Table S1 is based solely on the positions of the bands in the spectrum. We observe a good agreement with the calculated anharmonic spectra of FMISO for most of the bands, particularly in the fingerprint region. However, the harmonic bands at 786, 1121 and 1535 cm^−1^ were found to be in better agreement with the experimental band at 795, 1116 and 1534 cm^−1^. Such discrepancies can be due to intermolecular interactions but also to differences in the anharmonicity of the normal modes.

Next, we analyzed the calculated IR and Raman spectra (Figures S12–S16) for the five compounds in which the ^19^F atom was replaced by the ^18^F radioisotope. The calculations were performed for the most stable conformers in each case. The harmonic spectra for FMISO, FETNIM, FETA and FAZA exhibit a distinct ν(CF) mode at 977, 992, 997 and 991 cm^−1^. In all four cases, this band suffers a blue-shift of 5, 6, 11 and 9 cm^−1^ in the ^18^F spectra with respect to the ^19^F spectra. When comparing anharmonic frequencies, this difference decreases for FETNIM (3 cm^−1^), remains the same for FETA and increases for FMISO (6 cm^−1^) and FAZA (13 cm^−1^). No other significant differences were observed in the calculated harmonic bands of the IR and Raman spectra of these compounds.

In the case of EF5, spectroscopic calculations reveal the presence of multiple bands involving ν(CF) and ν(CF_2_) vibrations. Based on the results of the conformational analysis, we considered both conformers as the relative Boltzmann populations were very similar 55.05% for 1g and 44.95% for 2g. Upon substituting one fluorine atom with the ^18^F isotope we have not noticed any significant shifts in the ^18^F harmonic spectra for these bands in either conformer, as all differences were less than 5 cm^−1^.

To conclude this section, it is worth noting that all five investigated radiopharmaceuticals maintain the vibrational characteristics of the **2nim** template, both in terms of band positions and intensities. Furthermore, the computed anharmonic frequencies generally exhibit better agreement with experimental data. However, in some instances, the scaled harmonic frequencies have also proven to be useful for reliable assignments of vibrational spectra.

### 2.3. NMR Data

Given the lack of structural information for the radiopharmaceuticals, we compiled in this section the available experimental NMR data for the investigated compounds and compared the observed values with those predicted by quantum chemical calculations carried out at the PCM-B3LYP/6-311+G(d,p) level of theory (see Table 4, Table 5, Table 6, Table 7 and Table 8).

For all compounds the geometries have been fully optimized prior to NMR calculation at the same level of theory. Atom numbering schemes for all compounds are given in Figure 1. The computed NMR data were determined using the appropriate solvent specified in the respective available experimental works. Tetramethylsilane (TMS) was used as an NMR reference standard for FMISO (both for ^1^H and ^13^C spectra), FAZA (both for ^1^H and ^13^C spectra), FETA (for ^1^H spectrum), EF5 (for ^1^H spectrum) and for **2nim** (both for ^1^H and ^13^C spectra), whereas the CFCl_3_ or CF_3_COOH standards have been employed for ^19^F nuclei, according to the experimental conditions. The calculated spin-spin couplings are reported merely where experimental values are available. No scaling procedure was applied for any type of nuclei.

First, we have to note that in the case of the FETNIM compound, NMR experimental data have only been documented for one of its precursors, as reported by Yang et al. [18]. To our knowledge, NMR data for the compound itself are not available and it will be excluded from the following discussion. However, we have provided computational data for the chemical shifts and spin-spin couplings in Table S6 in the Supplementary Material for future reference. On the other hand, for the FMISO and FAZA compounds, a more substantial body of experimental NMR data has been amassed for the ^1^H, ^13^C and ^19^F nuclei and therefore allow for a more detailed analysis.

As seen in Table 4, Table 5, Table 6, Table 7 and Table 8, the overall agreement between the experimental and calculated NMR data is good, both for chemical shifts and J coupling constants. Significant discrepancies are evident for the chemical shifts associated with the ^13^C nuclei, which is a common behavior (see, for instance, ref. [19]) and it has not prevented the assignments for these nuclei. For the chemical shifts of the ^19^F nuclei, large deviations between experiment and theory have been observed (e.g., −230.70 ppm experimental vs. −266.16 ppm calculated, in DMSO for FMISO). In order to mitigate systematic errors for these nuclei, we employed a linear regression approach, a common practice found in the literature [20,21,22]. Thus, we fitted the calculated shifts against the experimental values (see Figure S17), resulting in the following equation for the scaled ^19^F chemical shifts at the B3LYP/6-311+G(d,p) level of theory:

(2) $$\delta_{scaled}(\mathrm{ppm})={0.90977\times \delta }_{predicted}(\mathrm{ppm})+10.71500$$

The goodness of fit is supported by the value of R^2^ = 0.9992, as well as the statistical parameters of mean absolute error (MAE = 1.486 ppm) and root mean square deviation (RMSD = 1.713 ppm).

Based on this scaling procedure, we can predict the chemical shifts for ^19^F nuclei in FETA and FETNIM at −225.08 and −232.69 ppm, respectively.

For FMISO, one can note a much lower value of the calculated chemical shift for the H19 nucleus with respect to its experimental counterpart (see Table 4). This discrepancy may be attributed to intermolecular hydrogen bonding (HB) interactions involving this particular atom. We note that the optimized conformer with intramolecular HB O12-H19-O7 still does not replicate successfully the observed chemical shift.

For each compound, *J* couplings have been predicted by calculations at least in qualitative agreement with experimental results. Largest deviations between experiment and theory are observed for ^1^*J*_C11H21_ and ^3^*J*_F13H18_ in case of FMISO, and ^2^*J*_F19C12_ and ^1^*J*_F19C18_ for FAZA.

**Table 4:** Experimental and calculated NMR data for FMISO.

Nucleus ^a^	Experimental Data ^b^		Calculated Data ^d^	
	Chemical Shift (ppm)	*J* Coupling (Hz)	Chemical Shift (ppm)	*J* Coupling (Hz)
H(14) H(15)	7.64, 7.22 *7.20*, *7.15*		7.44, 7.35	-
H(16)	4.00–4.10 *4.30*–*4.26*	n.a. ^c^	3.90	-
H(17)	4.65 *4.77*	13.70, 3.60 *13.00, 3.00*	4.76	^2^*J*_H17H16_ = 12.70 ^4^*J*_H17H19_ = 2.32
H(18)	4.48–4.58 *4.54*–*4.57*	n.a.	4.55	-
H(19)	5.64 *5.30*	n.a.	2.17	-
H(20) H(21)	4.32–4.46 *4.32*–*4.52*	n.a.	4.94, 4.89	-
C(2)	144.91	n.a.	154.60	-
C(4) C(5)	128.52, 127.35	n.a.	140.81, 136.10	-
C(9)	51.41	7.60	55.40	^3^*J*_C9H19_ = 8.26
C(10)	67.70	19.30	76.19	^3^*J*_C10F13_ = 15.15
C(11)	84.58	169.10	93.08	^1^*J*_C11H21_ = 147.9
F(13)	−230.70	46.00 19.00	−266.16	^2^*J*_F13H20_ = 46.46 ^2^*J*_F13H21_ = 49.90 ^3^*J*_F13H18_ = 12.79

^a^ Atom numbering can be seen in Figure 1. ^b^ from ref. [23] (in DMSO) and ref. [17] (in CDCl_3_). Experimental values in *italics* for ^1^H are from ref. [17]. ^c^ not available. ^d^ solvent: DMSO or CHCl_3_; reference standard: TMS for ^1^H and ^13^C, CFCl_3_ for ^19^F.

**Table 5:** Experimental and calculated NMR data for FAZA.

Nucleus ^a^	Experimental Data ^b^		Calculated Data ^d^	
	Chemical Shift (ppm)	*J* Coupling (Hz)	Chemical Shift (ppm)	*J* Coupling (Hz)
H(14)	7.79/*7.68*	1.00/*1.00*	7.72	^3^*J*_H14H15_ = 0.86
H(15)	7.28/*7.14*	1.00/*1.00*	7.43	^3^*J*_H15H14_ = 0.86
H(20)	6.48/*6.44*	1.00/*1.50*	6.40	^3^*J*_H20H21_ = 1.92
H(21)	4.13/*4.66*	2.10/*n.a*. ^c^	4.39	^3^*J*_H21H22_ = 3.48
H(22)	4.62/*4.50*	n.a./6.00, *2.00* *1.50*	4.79	^3^*J*_H22H23_ = 8.06 ^3^*J*_H22H21_ = 4.13 ^4^F_16H22_ = 2.50
H(23)	4.56/*4.60*	n.a./*n.a.*	4.30	-
H(26)	4.66/*4.20*	n.a./*57.00*	5.09	*J*_F19H26_ = 51.17
H(27)	4.66/*4.20*	n.a./*57.00*	4.87	*J*_F19H27_ = 47.22
C(4)	123.80/*125.22*	n.a./*125.22*	130.62	-
C(5)	126.90/*128.25*	n.a./*128.25*	137.12	-
C(9)	95.50/*96.80*	n.a./*n.a.*	104.38	-
C(10)	82.20/*83.63*	n.a./*n.a.*	94.92	-
C(11)	75.80/*77.21*	5.60/*4.70*	82.28	^3^*J*_F19C11_ = 6.54
C(12)	87.70/*89.04*	20.60/*20.60*	88.67	^2^*J*_F19C12_ = 13.61
C(18)	82.20/*83.63*	170.00/*169.70*	85.30	^1^*J*_F19C18_ = −212.43
F(19)	−227.20 ^d^/ *−147.72* ^e^	n.a./*n.a*.	−273.48 ^d^/ *−180.55* ^e^	-

^a^ Atom numbering can be seen in Figure 1. ^b^ from refs. [24] (in CD_3_OD) and [25] (in CD_3_OD); Experimental values in *italics* for ^1^H and ^13^C are from ref. [25]. ^c^ not available. ^d^ solvent: methanol; reference standards: TMS for ^1^H and ^13^C, CFCl_3_ and CF_3_COOH for ^19^F; ^e^ Values in italics for ^19^F refer to CF_3_COOH reference standard.

**Table 6:** Experimental and calculated NMR data for FETA.

Nucleus ^a^	Experimental Data ^b^		Calculated Data ^d^	
	Chemical Shift (ppm)	*J* Coupling (Hz)	Chemical Shift (ppm)	*J* Coupling (Hz)
H(14)	7.40	1.30	7.42	^3^*J*_H14H15_ = 0.81
H(15)	7.14	1.30	7.43	^3^*J*_H15H14_ = 0.81
H(18)	4.83	n.a. ^c^	<5.07>	-
H(19)				
H(21)	3.50	26.75	<3.75>	<^3^*J*_FH_>_trans_ = 27.09
H(22)		4.80		<^3^*J*_HH_>_cis_ = 5.71
H(23)	4.44	47.50	<4.70>	<^2^*J*_FH_> = 49.25
H(24)		4.80		<^3^*J*_HH_> = 5.71

^a^ Atom numbering can be seen in Figure 1. ^b^ from ref. [26]; ^c^ not available; ^d^ solvent: DMSO; reference standard: TMS, < > represents average values.

**Table 7:** Experimental and calculated NMR data for EF5.

Nucleus ^a^	Experimental Data ^b^		Calculated Data ^d^	
	Chemical Shift (ppm)	*J* Coupling (Hz)	Chemical Shift (ppm)	*J* Coupling (Hz)
H(14)	7.54	n.a. ^c^	7.63	-
H(15)	7.15	n.a.	7.43	-
H(23)	5.37	n.a.	<5.22>	-
H(24)				
H(25)	8.22	n.a.	7.14	-
H(26)	4.06	n.a.	<3.97>	-
H(27)				
F(21,22)	−81.70	n.a.	−102.33	-
F(18,19)	−118.76	16.00	−145.50	^3^J_FF_ = 19.15

^a^ Atom numbering can be seen in Figure 1. ^b^ from ref. [27]; ^c^ not available; ^d^ solvent: water, reference standards: TMS for ^1^H, CHCl_3_ for ^19^F; < > represents average values.

**Table 8:** Experimental and calculated NMR data for **2nim**.

Nucleus ^a^	Experimental Data ^b^		Calculated Data ^d^	
	Chemical Shift (ppm)	*J* Coupling (Hz)	Chemical Shift (ppm)	*J* Coupling (Hz)
H(9)	n.a.	n.a. ^c^	10.14	-
H(10) H11	7.41 *7.26*	n.a.	7.54	-
C(2)	146.10	n.a.	154.95	-
C(4) C(5)	126.35	n.a	<134.78>	-

^a^ Atom numbering can be seen in Figure 1. ^b^ from refs. [15,28]; Experimental values in *italics* for ^1^H are from ref. [28]. ^c^ not available; ^d^ solvent: DMSO; reference standard: TMS, < > represents average values.

### 2.4. TD-DFT Data

#### 2.4.1. 2-Nitroimidazole

In the following, we were interested in the electronic absorption properties of the compounds under examination. Particularly, we focused on calculating the absorption wavelengths and the nature of the involved transitions, as well as the electronic density changes triggered by the electronic excitation of these radiopharmaceuticals. For the following discussion, we considered only the electronic transitions with λ^abs^ ≥ 200 nm and f ≥ 0.01.

The three singlet-singlet electronic transitions of **2nim** in water, with oscillator strengths larger than 0.01, are listed in Table 9.

According to quantum chemical calculations, the transition to the first singlet excited state has a very low oscillator strength and the λ_max_ band in the UV–Vis, predicted at 315 nm, is mainly due to the HOMO → LUMO excitation with a contribution of 99.12%. The other two significantly intense bands appear as a result of S0 → S5 and S0 → S5 transitions (see Table 9).

The low probability of the S0 → S1 excitation is due to a poor overlap of the orbitals involved in this electronic transition, and consequently, a low transition dipole moment. The corresponding natural transition orbitals (NTOs) [29] HOTO and LUTO for this transition are depicted in Figure S18. On the other hand, for the transition to the second excited state the HOTO and LUTO NTOs suggest a good overlap, particularly on the nitro group, which confirms the high intensity of the S0 → S2 excitation.

It is worth mentioning that the transition to the lowest excited state does not involve the HOMO → LUMO excitation for **2nim**, while for its derivatives, the same excitation contributes to a lesser extent. Such behaviors have been noted very recently by Kimber and Plasser [30]. These authors concluded that the interplay of the terms corresponding to the dynamic electron−hole binding energy, exchange repulsion between electron and hole and/or to the response of the exchange-correlation potential govern the different types of excited states like locally excited or charge-transfer character and influence the pairs of occupied and unoccupied molecular orbitals involved in particular transitions.

To the best of our knowledge, there are no experimental UV–Vis data for **2nim**, but Yu and Bernstein [31] reported such data for the **4nim** isomer.

To validate the computed data for **2nim**, we also calculated the absorption spectrum of **4nim**, being able to quantitatively reproduce the experimental data. That is, the three main peaks observed at 311, 287 and 203 nm were calculated at 300, 274 and 201 nm, respectively. Our calculated data for **4nim** agree very well with the computationally predicted values at the CASSCF(10,7)/6-31G(d) level of theory [31]. Based on the agreement between experiment and theory for **4nim**, we estimate that the calculated data for **2nim** will also demonstrate a high degree of reliability, thus providing confidence in the validity of our computational results for this compound.

The PBE0 [32] and ωB97XD [33] density functionals are also frequently employed for the calculation of electronic transitions. Both predict UV–Vis spectra shapes similar to that obtained by using the B3LYP functional, with zero intensity for the S0 → S1 transition. Within the 200–350 nm wavelength range, these functionals yield band positions at 306 nm, 234 nm and 200 nm for PBE0 and 293 nm, 222 nm and 197 nm for ωB97XD, respectively.

#### 2.4.2. 2-Nitroimidazole Derivatives

**Figure 4:**
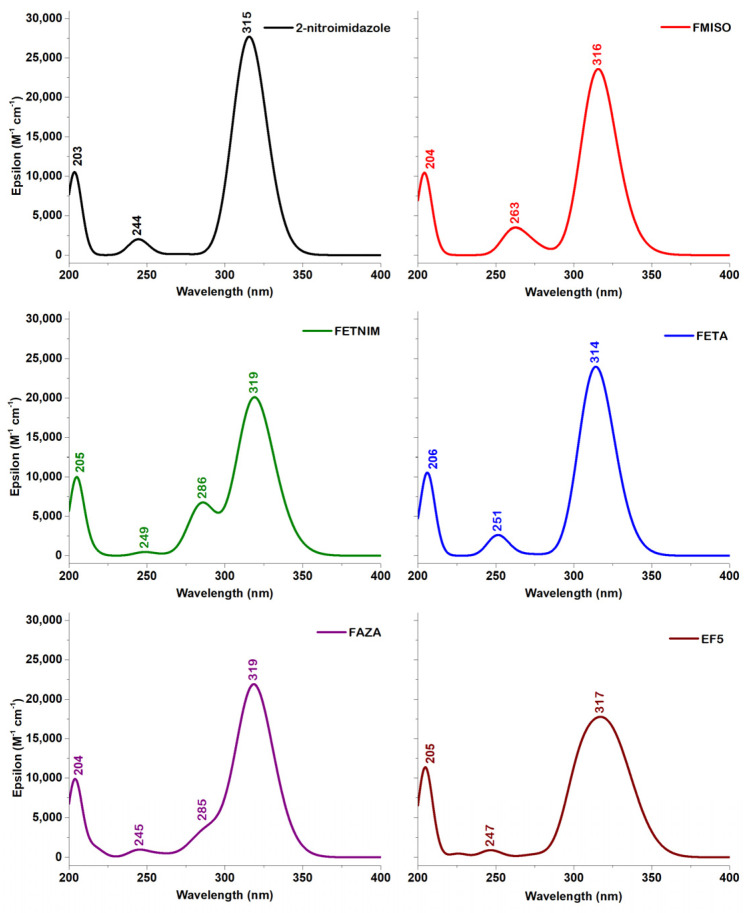
Calculated UV–Vis spectra for **2nim** and the five derivatives at the PCM(H_2_O)-B3LYP/6-311+G(d,p) level of theory.

As shown in Figure 4, the calculated λ_max_ bands for the derivatives display remarkable similarity, with each of them closely following the pattern observed for **2nim**.

Table 10 lists the calculated excitation energies of the most stable conformers in water for the five 2-nitroimidazole derivatives. In contrast to the results obtained for the **2nim** molecule, the transition to the S1 excited singlet state is allowed for all five compounds. Furthermore, the largest contributions to the S1 excited state are HOMO-2 → LUMO (60%) predicted at 331 nm for FMISO, HOMO-3 → LUMO (59%) at 332 nm for FETNIM, HOMO-4 → LUMO (58%) at 328 nm for FAZA, HOMO-1 → LUMO (78%) at 328 nm for FETA and HOMO-3 → LUMO (41%) at 335 nm for EF5. The contribution of the HOMO → LUMO transition to the S1 excited state is less than 10% in each case.

As seen in Table 10, the largest contribution for the S2 singlet excited state, corresponding to the most intense band, is due to the HOMO → LUMO transitions for all five compounds: 89% for FMISO predicted at 315 nm, 76% for FETNIM at 318 nm, 73% for FAZA at 317 nm, 60% for FETA at 323 nm and 54% for EF5 at 322 nm.

For FMISO, the band predicted at 316 nm appears as a result of the joint contributions of the transitions to the S1 and S2 excited states. For FETNIM and FAZA, the λ_max_ bands at 319 nm are again attributed to the transitions to the S1 and S2 states. However, for FETNIM we observe an additional band at 286 nm, while for FAZA a shoulder is seen at 297 nm. They are assigned to the transitions to the S3 excited states with the main contributions being HOMO-1 → LUMO, 92.78% for FETNIM and HOMO-2 → LUMO, 68.72%, for FAZA (see Table 10).

The band at 314 nm in the spectrum for FETA is due to the joint transitions to the S2, S3 and S4 states. In contrast to the other investigated compounds where the S2 transition was calculated with the highest oscillator strength, the transition to the S4 excited state of FETA, predicted at 312 nm, has the largest value for the oscillator strength and is mainly due to HOMO → LUMO (54.27%) and HOMO-1 → LUMO (37.03%) transitions.

A distinct characteristic for the UV–Vis spectrum of EF5 is the broadness of the 317 nm band. The main contributions to it comes from transitions to the S2 and S3 states, predicted at 322 and 307 nm, respectively, and mainly due to the HOMO → LUMO (for S2) and HOMO-1 → LUMO (for S3) transitions.

**Figure 5:**
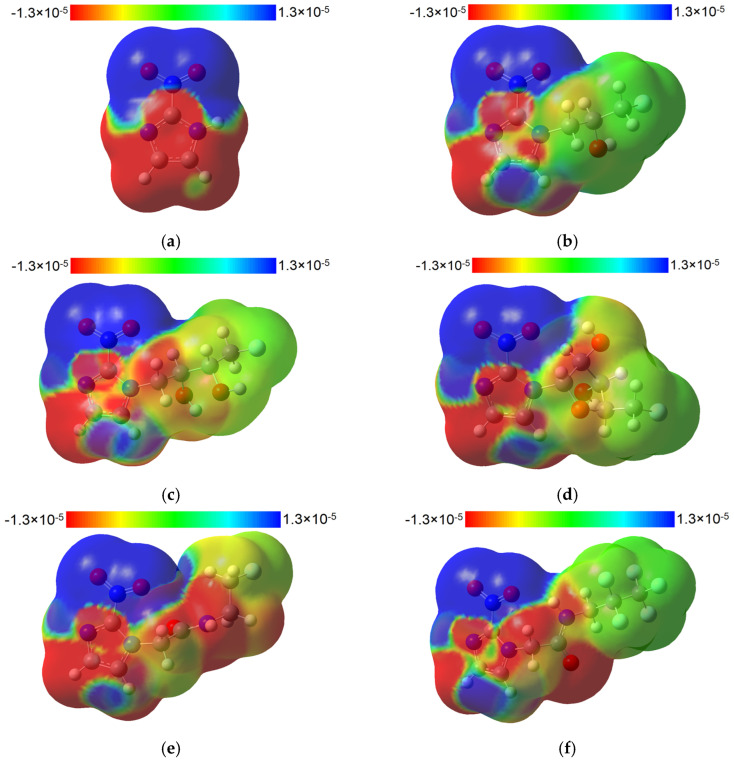
The total SCF electronic density isosurfaces (0.02 a.u.) mapped with electronic density differences between the S2 singlet excited state and the ground state for **2nim** (**a**) and between the S1 singlet excited state and the ground state for FMISO (**b**), FETNIM (**c**), FAZA (**d**), FETA (**e**) and EF5 (**f**), calculated at the B3LYP/6-311+G(d,p) level of theory in water. The blue (red) areas denote an increase (depletion) in electronic density in the excited state.

An elegant and useful way to describe how the electronic density changes as a result of excitation is by plotting the difference between the electronic densities of the two states involved in the transition. In Figure 5 are illustrated the electronic density differences (EDDs) between the state of interest for **2nim** and the five derivatives. In the case of **2nim**, we note a charge transfer from the imidazole ring to the nitro group during the transition to the excited state. All the other derivatives show basically the same pattern for EDDs, excepting the FETA and EF5 compounds for which the carbonyl group acts together with the imidazole group as a donor of electronic density to the nitro group.

## 3. Materials and Methods

For this work, we used state-of-the-art, yet confident, density functional theory (DFT) methods to obtain the calculated properties of the investigated compounds. All calculations were performed with the Gaussian 16, revision C.01 software package [34] by using the B3LYP hybrid exchange-correlation functional [35,36,37,38] in conjunction with Pople’s style split-valence basis sets [39].

Tight and very tight criteria were employed to define the convergence of the molecular geometries and SCF convergence. The ultrafine grid was applied for the numerical integration of the electronic density. All geometry optimizations were followed by vibrational frequency calculations in order to check and validate the minima on the potential energy surfaces (PES) of the investigated molecules. Gibbs free energies were calculated using the standard thermochemistry model (T = 298.15 K).

Relaxed PESs in the gas phase were calculated at the B3LYP/3-21G level of theory. Geometry optimization and IR and Raman spectra calculations were performed at the B3LYP/6-311+G(d,p) level of theory for all the minima found on the PES. Solvent effects in water have been accounted for by using the implicit polarizable continuum model [40] within the integral equation formalism [41]. We chose water as a solvent because radiopharmaceuticals are usually administered intravenously to patients in an aqueous saline solution.

Both harmonic and anharmonic approximations were used to calculate the IR and Raman spectra. The harmonic spectra were scaled by a factor of 0.9668 [42], and the anharmonic wavenumbers were calculated using the vibrational second-order perturbation theory (VPT2) [43], as implemented in the Gaussian software package. The anharmonic IR and Raman spectra were simulated using the computed anharmonic wavenumbers. The IR intensities and Raman activities were those obtained within the harmonic approximation. Because of their very low calculated intensities, the overtones and the combination modes were not included in the simulation.

This approach was chosen because the anharmonic calculations can result in spurious very large activities [44,45]. Pure Lorentzian band shapes with a half-width at half maximum of 4 cm^−1^ were used for the plots of the calculated IR and Raman spectra.

NMR spectra calculations were performed using the GIAO (gauge-including atomic orbital) method [46,47] as implemented in the Gaussian software package, at the B3LYP/6-311+G(d,p) level of theory. The computed NMR shielding tensors were converted into chemical shifts using the standard referencing method to tetramethylsilane (TMS) for ^1^H and ^13^C, and to trichloro-fluoro-methane (CFCl_3_) for ^19^F. The TMS and CFCl_3_ shielding tensors were calculated at the same level of theory as that used for the **2nim** derivatives, after geometry optimization of the reference molecules.

To assess the absorption properties of the compounds, time-dependent density functional theory (TD-DFT) methods [48] were employed, as implemented in the Gaussian software package. Single-point calculations on the excitation energies to singlet excited states were carried out at the TD-B3LYP/6-311+G(d,p) level of theory. The simulated UV–Vis spectra were obtained by convoluting the calculated vertical transitions with Gaussian functions with a half-width at half maximum of 0.15 eV.

## 4. Conclusions

The present study offers a detailed structural investigation of the most successful PET imaging agents for evaluating tumor hypoxia based on **2nim**. To our knowledge, this is one of the very few works available in the current literature that provide a thorough characterization of the electronic structures and spectroscopic properties of radiopharmaceuticals.

Conformational analysis, vibrational and NMR spectra were obtained by means of quantum chemical DFT calculations. We have determined the structures of all unique conformers in the gas phase and water. Apart from the EF5 compound, important changes are observed in the conformers’ stabilities when going from the gas phase to the liquid state for all the other compounds. The available experimental data were accurately reproduced by the computational results obtained in this work. Anharmonic calculations proved to be more consistent in predicting the experimental IR spectrum for **2nim**. Both calculated chemical shifts and spin-spin couplings were in good agreement with the available experimental data.

The photophysical properties of the compounds were predicted by means of TD-DFT calculations. UV–Vis spectra were obtained for all investigated compounds; however no such experimental data were available for validation. The migration of the electronic density during transitions was explained based on the electronic density differences between the ground and the excited states.

Our findings contribute to a deeper understanding of the spectroscopic properties of the five investigated radiopharmaceuticals. Moreover, the obtained results in this study can prove useful in further understanding the receptor binding mechanism of current and prospective 2-nitroimidazole based radiopharmaceuticals. Computational data indicate that the electronic excitation energies of all examined compounds fall outside the NIR spectral range, rendering them unsuitable as optical imaging probes.

In the development of multimodal agents based on 2-nitroimidazole, particularly for applications in nuclear medicine and optical imaging/photodynamic therapy, it would be imperative to incorporate a functional group that is compatible with both radiofluorination and the requisite absorption/emission characteristics. This dual functionality is essential to ensure the efficacy and versatility of these agents in their respective diagnostic and therapeutic applications.

## Figures and Tables

**Table 1:** Dihedral angles used to generate the relaxed PESs for the five investigated molecules.

Compound	Dihedral Angle (°)
FMISO	C4-N3-C9-C10
	N3-C9-C10-C11
FETNIM	C4-N3-C9-C10
	N3-C9-C10-C11
FAZA	C4-N3-C9-C10
FETA	C4-N3-C9-C10
	N3-C9-C10-N11
EF5	C4-N3-C9-C10
	N3-C9-C10-N11

**Table 2:** Calculated relative free energies and Boltzmann populations of conformers in the gas phase and water, at the B3LYP/6-311+G(d,p) level of theory.

Compound	Gas Phase Conformer	ΔG (kcal∙mol^−1^)	Relative Boltzmann Population (%)	Water Conformer	ΔG (kcal∙mol^−1^)	Relative Boltzmann Population (%)
FMISO	1g	0.000	62.15	1w	0.000	65.91
	2g	0.630	21.45	2w	0.690	20.56
	3g	1.030	10.92	3w	1.280	7.59
	4g	1.800	2.97	4w	1.720	3.61
	5g	1.900	2.51	5w	1.980	2.33
FETNIM	1g	0.000	71.36	1w	0.000	68.26
	2g	1.030	12.53	2w	0.640	23.16
	3g	1.280	8.22	3w	1.370	6.75
	4g	1.660	4.33	4w	2.780	0.62
	5g	1.990	2.48	5w	2.810	0.59
	6g	2.480	1.08	6w	2.820	0.58
	7g	5.390	0.01	7w	4.850	0.02
FAZA	1g	0.000	99.25	1w	0.000	69.66
	2g	3.190	0.45	2w	0.530	28.46
	3g	3.450	0.29	3w	2.140	1.88
FETA	1g	0.000	50.84	1w	0.000	63.97
	2g	0.020	49.16	2w	0.340	36.03
EF5	1g	0.000	54.21	1w	0.000	55.05
	2g	0.100	45.79	2w	0.120	44.95

**Table 3:** B3LYP/6-311+G(d,p) calculated wavenumbers of **2nim** in the gas phase alongside the corresponding wavenumbers of the same modes in the five derivative compounds.

Mode	Theoretical Wavenumbers in Gas Phase (cm^−1^)								Assignments ^c^
	2nim			FMISO 1g	FETNIM 1g	FETA 1g	FAZA 1g	EF5 1g	
	Harmonic	Anharmonic	Experimental ^a^						
Q1	201	199	n.a. ^b^	216	187	174	200	195	oop. def. (**2nim**)
Q2	242	240	n.a.	244	298	308	285	306	*ρ*(**2nim**)
Q3	548	539	n.a.	566	562	540	600	546	δ(CNO)
Q4	600	572	623	638	635	625	631	628	γ(imidazole)
Q5	660	645	649	660	660	660	661	660	γ(imidazole)
Q6	777	772	798	785	785	789	798	788	γ(CH)_imidazole_ (ip.)
Q7	840	830	820	855	854	857	866	857	δ(NO_2_) + δ(CN_2_)_imidazole_
Q8	890	872	n.a.	888	886	893	893	894	γ(CH)
Q9	929	916	944	932	926	924	934	922	δ(CNC) + δ(NCC)
Q10	997	984	985	-	-	-	-	-	δ(NCN) + δ ν(C-NO_2_) + (CH)
Q11	1056	1067	n.a.	-	-	-	-	-	β(CH) + β(NH)
Q12	1083	1102	1103	1068	1061	1059	1047	1167	δ(CH)
Q13	1150	1158	1155	1156	1154	1151	1156	1153	δ(CN_2_) + β(CH)
Q14	1217	1223	1269	1270	1256	1260	1224	1260	β(CH) + ν_sym_(NO_2_) + ip. def.(imidazole)
Q15	1330	1342	1348	1325	1323	1323	1324	1318	ν(C-N)_imidazole_ + δ(NO_2_) + β(CH, NH)
Q16	1333	1349	1376	1331	1332	1329	1337	1331	ν(C-NO_2_) + β(CH) + δ(NO_2_)
Q17	1386	1393	1425	1384	1380	1377	1380	1377	ν(C-N)_imidazole_ + β(NH) + β(CH)
Q18	1456	1467	1494	1464	1463	1459	1445	1459	ν(C-N)_imidazole_ + β(NH) + β(CH)
Q19	1494	1513	1520	1476	1474	1473	1467	1474	ν(C=N)_imidazole_ + ν(C=C) + β(NH) + β(CH)
Q20	1546	1559	1554	1535	1533	1536	1530	1538	ν_asym_(NO_2_)
Q21	3141	3123	3146	3139	3138	3140	3140	3140	ν_asym_(CH)
Q22	3165	3147	3164	3166	3164	3160	3189	3161	ν_sym_(CH)
Q23	3512	3469	3423	-	-	-	-	-	ν(NH)

^a^ from references [15,16]; ^b^ not available. ^c^ oop.—out-of-plane; ip.—in plane; def.—deformation; *ρ*—rocking; ν—stretching; β—in plane bending XYH angles; γ—out-of-plane bending; δ—in-plane bending; sym.—symmetric; asym—antisymmetric.

**Table 9:** Theoretical UV–Vis absorption spectral data for **2nim** in water at the PCM(H_2_O)-TD-B3LYP/6-311+G(d,p) level of theory.

Compound	Excited State *	$\lambda^{abs}(nm)$	f	Transitions	Contributions (%)
	S2	315	0.3287	H→L	99.12
	S5	245	0.0239	H-2→L	90.08
	S6	204	0.1247	H-4→L	87.57

* Only singlet-singlet transitions with λ^abs^ ≥ 200 nm and f ≥ 0.01; H—HOMO, L—LUMO.

**Table 10:** Theoretical UV–Vis absorption spectral data for FMISO, FETNIM, FAZA, FETA and EF5 in water at the PCM(H_2_O)-B3LYP/6-311+G(d,p) level of theory.

Compound	Excited State *	$\lambda^{abs}(nm)$	f	Transitions	Contributions (%)
FMISO 1w	S1	331	0.0221	H-4 → L	17.34
				H-2 → L	59.61
				H-1 → L	10.33
	S2	315	0.2708	H → L	88.58
	S5	270	0.0126	H-4 → L	61.04
				H-1 → L	22.37
	S6	260	0.0332	H-3 → L	80.51
	S8	204	0.1194	H-5 → L	78.21
FETNIM 1w	S1	332	0.0399	H-5 → L	14.87
				H-3 → L	59.22
				H → L	18.96
	S2	318	0.2172	H-3 → L	17.28
				H → L	76.24
	S3	286	0.0784	H-1 → L	92.78
	S9	205	0.1166	H-6 → L	82.94
FAZA 1w	S1	328	0.0536	H-4 → L	58.31
				H → L	22.26
	S2	317	0.2195	H-4 → L	21.16
				H → L	73.38
	S3	297	0.0248	H-4 → L	22.66
				H-2 → L	68.72
	S4	286	0.0238	H-4 → L	54.06
				H-3 → L	15.42
				H-2 → L	20.91
	S10	204	0.1169	H-9 → L	23.39
				H-7 → L	64.87
FETA 1w	S2	323	0.0541	H-1 → L	59.96
				H → L	30.57
	S3	314	0.0508	H-2 → L	84.74
				H → L	13.82
	S4	312	0.1961	H → L	54.27
				H-1 → L	37.03
	S7	251	0.0312	H-4 → L	92.54
	S10	206	0.1221	H-6 → L	84.89
EF5 1w	S1	335	0.0525	H-5 → L	12.02
				H-3 → L	41.36
				H → L	27.89
	S2	322	0.1329	H-3 → L	23.75
				H → L	54.16
	S3	307	0.0874	H-1 → L	72.79
				H → L	12.56
	S4	302	0.0547	H-2 → L	75.39
	S10	205	0.1299	H-7 → L	11.53
				H-6 → L	77.38

* Only singlet-singlet transitions with λ^abs^ ≥ 200 nm and f ≥ 0.01; H—HOMO, L—LUMO.

## Data Availability

Data are contained within the article and Supplementary Materials.

## Supplementary Materials

The following supporting information can be downloaded at: https://www.mdpi.com/article/10.3390/molecules29071505/s1, Figure S1: optimized structures of the conformers for FMISO, FETNIM, FAZA, FETA and EF5; Figures S2–S6: Harmonic IR and Raman spectra for the most stable conformers; Figure S7–S11: Harmonic and anharmonic IR and Raman spectra for the most stable conformers, Figures S12–S16 Harmonic IR and Raman spectra for the ^18^F variant of the derivatives; Figure S17: Fit of the calculated vs. experimental values for the chemical shifts of the ^19^F nuclei; Figure S18: NTOs corresponding to the first to excited singlet states of **2nim**; Tables S1–S5: Assignment of vibrational modes for the 5 derivatives; Table S6: Calculated NMR data for FETNIM; Tables S7–S12: Cartesian coordinates for the optimized structures of **2nim**, FMISO, FETNIM, FETA, FAZA and EF5.
