# Supplementary material for: Exploring Geometrical, Electronic and Spectroscopic Properties of 2-Nitroimidazole-Based Radiopharmaceuticals via Computational Chemistry Methods
Source: Molecules. 2024 Mar 28;29(7):1505. doi: 10.3390/molecules29071505 (PMC11013577; doi:10.3390/molecules29071505)
Supplement: Supplementary file 1 [file molecules-29-01505-s001.zip › molecules-2895641-supplementary.pdf]

molecules

MDPI

Supplementary material

Exploring Geometrical, Electronic and Spectroscopic Properties of 2-Nitroimidazole-Based Radiopharmaceuticals via Computational Chemistry Methods

George Crișan [1,2], Ștefan Stan [1], Vasile Chiș [1,3,*]

[1] Faculty of Physics, Babeș-Bolyai University, Str. M. Kogălniceanu 1, RO-400084 Cluj-Napoca, Romania; vasile.chis@ubbcluj.ro
[2] Department of Nuclear Medicine, County Clinical Hospital, Clinicilor 3-5, RO-400006 Cluj-Napoca, Romania; george.crisan@scjucluj.ro
[3] Institute for Research, Development and Innovation in Applied Natural Sciences, Babeș-Bolyai University, Str. Fântânele 30, RO-400327 Cluj-Napoca, Romania;
* Correspondence: vasile.chis@ubbcluj.ro

The authors will readily provide any additional computational data not included in the article or SI material to interested parties.

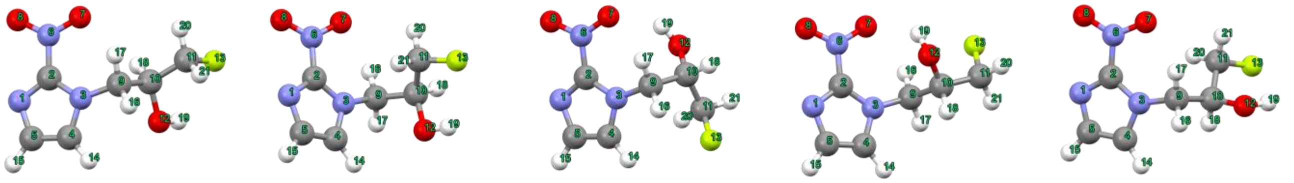

1g 2g 3g 4g 5g

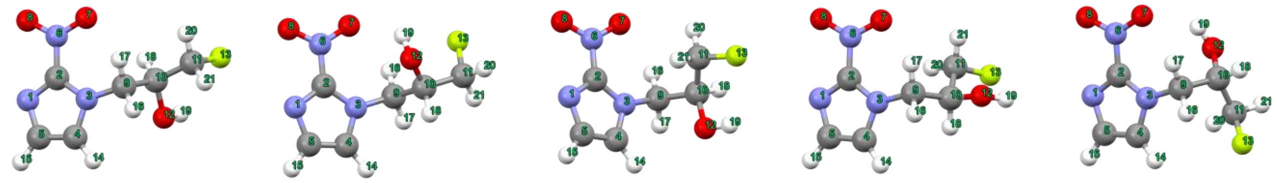

1w 2w 3w 4w 5w

(a)

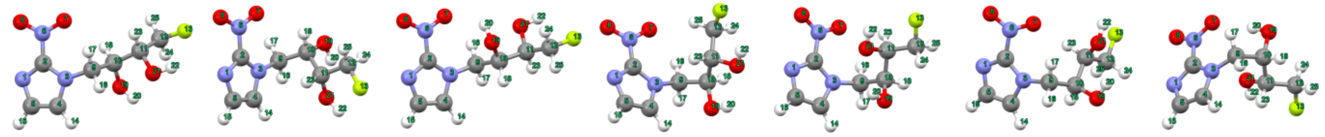

1g 2g 3g 4g 5g 6g 7g

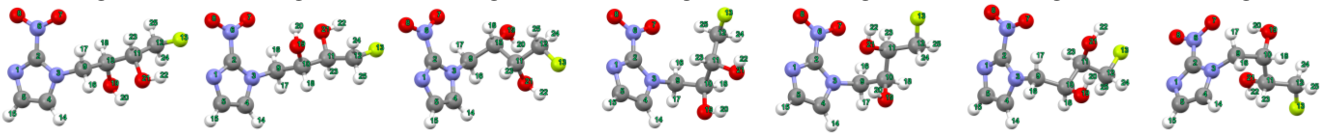

1w 2w 3w 4w 5w 6w 7w

(b)

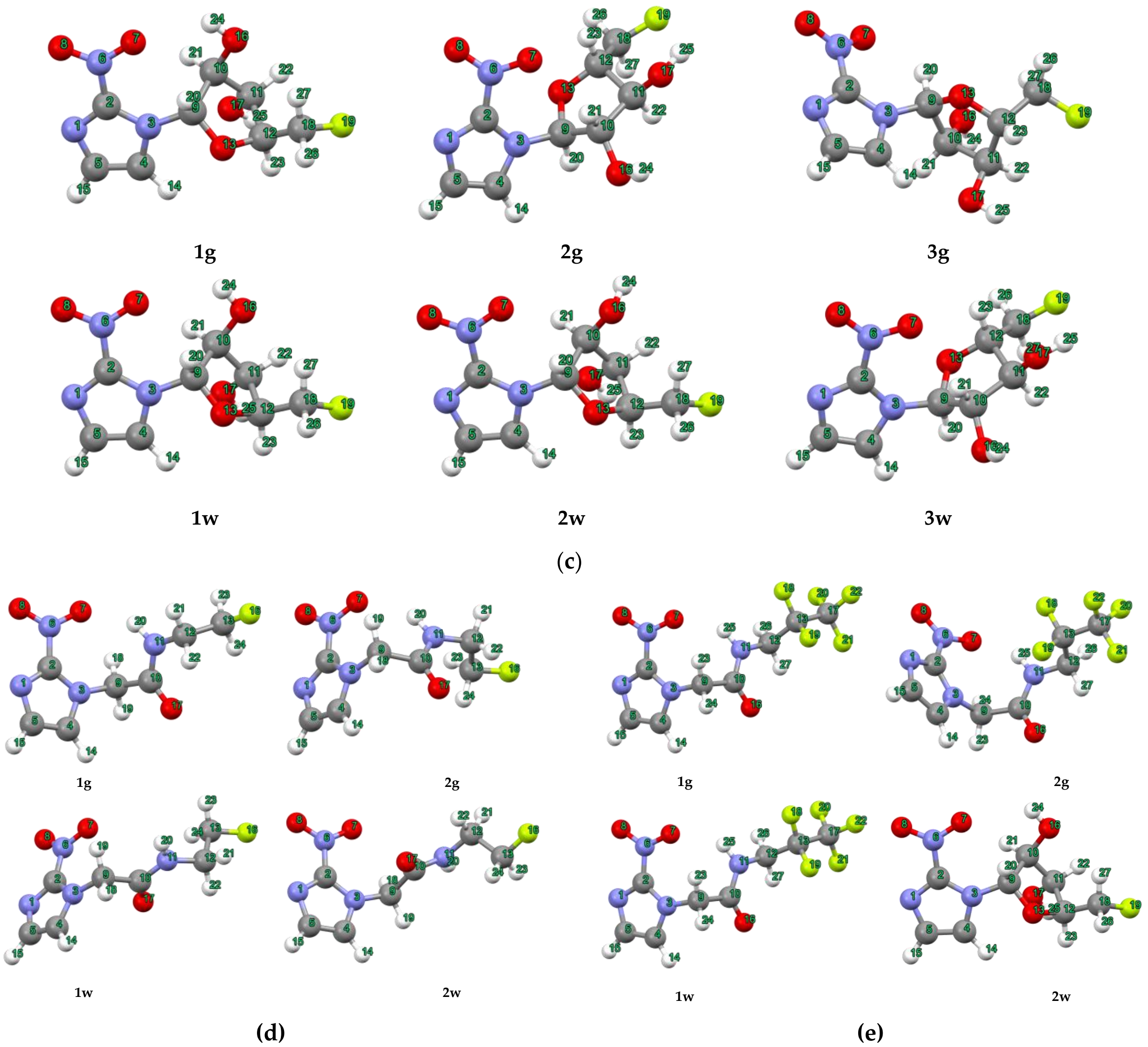

Figure S1. B3LYP/6-311+G(d,p) optimized structures of the conformers for (a) FMISO, (b) FETNIM, (c) FAZA, (d) FETA, (e) EF5, found as minima on the B3LYP/3-21G calculated 2D PES.

Table S1. B3LYP/6-311+G(d,p) calculated wavenumbers of FMISO in gas-phase.

Mode	Theoretical wavenumbers cm^{-1}			Assignment[b]
	Harmonic scaled	Anharmonic	Experimental Ref[a]	
Q1	216 (1g)	213		oop. def. (2nim)
Q2	244 (1g)	236		ρ(2nim)
Q3	386 (1g)	445		γ(fluropropan-2-ol)
Q4	637 (all)	634		γ(imidazole)
Q5	747 (1g+3g)	739		$\omega(NO_2)+\omega(CH)$
Q6	786 (1g)	778	795	$\omega(CH)_{imidazole}$
Q7	854 (all)	841	834	$\beta(NO_2)+\beta(CN_2)_{imidazole}$
Q8	882 (1g)	857		ν(CO)+γ(CH)
Q9	932 (all)	917	919	δ(CNC)+δ(NCC)
Q10	977 (1g+2g)	978		ν(CF)
Q11	1086 (1g)	1089		δ(CCC)
Q12	1121 (1g+3g)	1138	1116	τ(CH2)+ϱ(CH2)+δ(CH)
Q13	1156 (all)	1167	1161	$\beta(CN_2)+\beta(CH)$
Q14	1240 (1g+3g)	1251		$\gamma(CH_2)+\gamma(CH)+\beta(OH)$
Q15	1258 (2g)			$\gamma(CH_2)+\gamma(CH)+\beta(OH)$
Q16	1271 (1g)	1280	1279	$\beta(CH)+\nu_{sym}(NO_2)$+ip. def.(imidazole)
Q17	1280 (1g)	1305		ω(CH2)+ ω(CH)+ ω(OH)
Q18	1325 (all)	1333		$\nu(CN)_{imidazole} + \delta(NO_2) + \beta(CH, NH)$
Q19	1349	1363	1364	ω(CH2)
Q20	1390 (1g+2g)	1402		ω(CH2)+ ω(CH)+ ω(OH)
Q21	1421 (all)	1432		δ(CH2)
Q22	1464 (all)	1476		$\nu(CN)_{imidazole}+\beta(CH_2)+\beta(CH)$
Q23	1476	1490	1482	$\nu(CN)_{imidazole}+\nu(CC)+\beta(CH)$
Q24	1535 (all)	1553	1534	$\nu_{asym}(NO_2)$
Q25	2892 (2g+3g)			$\nu_{sym}(CH_2)$
Q26	2948 (1g)	2958		$\nu_{sym}(CH_2)$
Q27	2980 (all)	2959		$\nu_{sym}(CH_2)$
Q28	3007 (1g)	2973		$\nu_{asym}(CH_2)$
Q29	3139 (all)	3121	3104	$\nu_{asym}(CH)$
Q30	3166 (all)	3149	3255	$\nu_{sym}(CH)$
Q31	3621 (3g) / 3696 (1g)	3641		ν(OH)

[a] from ref [17].
[b] oop. – out-of-plane; ip. – in plane; def. – deformation; ρ - rocking; ν - stretching; β - in plane bending XYH angles; γ - out-of-plane bending; ω - wagging; δ - in-plane bending; sym. – symmetric; asym – antisymmetric.

Table S2. B3LYP/6-311+G(d,p) calculated wavenumbers of FETNIM in gas-phase.

Mode	Theoretical wavenumbers cm^{-1}		Assignment[a]
	Harmonic scaled	Anharmonic	
Q1	187 (all)	185	oop. def. (2nim)
Q2	244 (all)	238	γ(nitroimidazole)+ γ(fluorobutan)
Q3	405 (1g)	371	γ(fluorobutan)
Q4	531 (all)	520	τ(fluorobutan)
Q5	690 (1g+3g)	679	δ(CCO)
Q6	775 (1g+3g)	762	$\omega(CH)_{imidazole}$
Q7	855 (1g+3g)	843	$\beta(NO_2)+\beta(CN_2)_{imidazole}$
Q8	874 (1g)	856	$\nu(CO)+\tau(CH_2)$
Q9	884 (3g)		$\nu(CO)+\tau(CH_2)$
Q10	926 (1g+3g)	913	δ(CNC)+δ(NCC)
Q11	992 (all)	968	ν(CF)
Q12	1019 (1g+2g)	1029	ν(CC)
Q13	1096 (1g+2g)	1102	$\nu(CO)+\tau(CH_2)$
Q14	1161 (1g+2g)	1180	$\beta(CH)_{imidazole}$
Q15	1256 (all)	1269	$\beta(CH)+\nu_{sym}(NO_2)$+ip. def.(imidazole)
Q16	1288 (1g+2g)	1297	δ(OH)
Q17	1323 (all)	1333	$\nu(CN)_{imidazole}+\delta(NO_2)+\beta(CH)$
Q18	1375 (1g+2g)	1387	$\delta(OH)+\omega(CH_2)$
Q19	1426 (1g+2g)	1435	$\beta(CH_2)$
Q20	1463 (all)	1474	$\nu(CN)_{imidazole}+\beta(CH_2)+\beta(CH)$
Q21	1534 (all)	1550	$\nu_{asym}(NO_2)$
Q22	2914 (1g+2g)	2893	ν(CH)
Q23	2964 (all)	2973	$\nu_{sym}(CH_2)$
Q24	3138 (all)	3120	$\nu_{asym}(CH)$
Q25	3614 (all)	3575	$\nu_{sym}(CH)$
Q26	3689 (all)	3640	ν(OH)

[a] oop. – out-of-plane; ip. – in plane; def. – deformation; ρ - rocking; ν - stretching; β - in plane bending XYH angles; γ - out-of-plane bending; ω - wagging; δ - in-plane bending; sym. – symmetric; asym – antisymmetric.

Table S3. B3LYP/6-311+G(d,p) calculated wavenumbers of FAZA in gas-phase.

Mode	Theoretical wavenumbers cm^{-1}		Assignment
	Harmonic scaled	Anharmonic	
Q1	164	159	oop. def. (2nim)+oop. def. (oxolane)
Q2	200	193	oop. def. (2nim)
Q3	247	237	γ(oxolane)
Q4	394	385	γ(nitroimidazole) + β(oxolane)
Q5	554	546	γ(oxolane)
Q6	661	651	γ(imidazole)
Q7	733	721	δ(COC)
Q8	816	802	ω(CCO)
Q9	866	851	β(NO_2)+β(CN_2)$_{imidazole}$
Q10	935	923	δ(CNC)+δ(NCC)
Q11	991	1000	ν(CF)
Q12	1018	1026	ν(CO)
Q13	1073	1076	ν(CO) + β(CH)
Q14	1156	1169	β(CN_2)+β(CH)
Q15	1224	1236	β(CH)+ν_{sym}(NO_2)+ip. def.(imidazole)+β(OH)
Q16	1292	1304	β(OH)+β(CH)
Q17	1324	1338	ν(CN)$_{imidazole}$+ν_{sym}(NO_2) +β(CH
Q18	1380	1391	ν(CN)$_{imidazole}$+β(CH)
Q19	1445	1457	ν(CN)$_{imidazole}$+β(CH)
Q20	1530	1546	ν_{asym}(NO_2)
Q21	2965	2899	ν_{sym}(CH_2)
Q22	3025	2989	ν_{asym}(CH_2)
Q23	3140	3121	ν_{asym}(CH)
Q24	3189	3170	ν_{sym}(CH)
Q25	3674	3617	ν(OH)
Q26	3710	3668	ν(OH)

[a] oop. – out-of-plane; ip. – in plane; def. – deformation; ρ - rocking; ν - stretching; β - in plane bending XYH angles; γ - out-of-plane bending; ω - wagging; δ - in-plane bending; sym. – symmetric; asym – antisymmetric.

Table S4. B3LYP/6-311+G(d,p) calculated wavenumbers of FETA in gas-phase.

Mode	Theoretical wavenumbers cm^{-1}		Assignment[a]
	Harmonic scaled	Anharmonic	
Q1	174 (all)	168	oop. def. (2nim)
Q2	281 (all)	275	β(fluoroethyl)
Q3	322 (all)	319	β(fluoroethyl) + β(nitroimidazole)
Q4	491 (2g)		γ(fluoroethyl)
Q5	503 (1g)	496	γ(fluoroethyl)
Q6	590 (all)	532	γ(fluoroethyl)+ β(nitroimidazole)
Q7	650 (1g)	666	γ(fluoroethyl) + γ(nitroimidazole)
Q8	658 (2g)		γ(fluoroethyl) + γ(nitroimidazole)
Q9	791 (all)	764	γ(CH)imidazole + ϱ(CH_2)
Q10	998 (all)	1005	ν(CF)
Q11	1151 (all)	1164	β(CN_2)+β(CH)
Q12	1260 (all)	1193	β(CH)+ν_{sym}(NO_2)+ip. def.(imidazole)
Q13	1317 (all)	1320	ν(CN)+ω(CH_2)
Q14	1459 (all)	1472	$\nu(CN)_{imidazole}$+β(CH_2)+β(CH)
Q15	1514 (all)	1515	$\nu(CN)_{fluoroethyl}$
Q16	1536 (all)	1554	ν_{asym}(NO_2)
Q17	1695 (all)	1720	ν(C=O)
Q18	2948 (all)	2889	ν_{sym}(CH_2)
Q19	2966 (all)	2969	ν_{sym}(CH_2)
Q20	3024 (all)	2991	ν_{asym}(CH_2)
Q21	3140 (all)	3121	ν_{asym}(CH)
Q22	3445 (1g)	3388	ν(NH)
Q23	3452 (2g)		ν(NH)

[a] oop. – out-of-plane; ip. – in plane; def. – deformation; ρ - rocking; ν - stretching; β - in plane bending XYH angles; γ - out-of-plane bending; ω - wagging; δ - in-plane bending; sym. – symmetric; asym – antisymmetric.

Table S5. B3LYP/6-311+G(d,p) calculated wavenumbers of EF5 in gas-phase.

**Mode**	**Theoretical wavenumbers cm^{-1}**		**Assignment[a]**
	**Harmonic scaled**	**Anharmonic**	
Q1	195 (1g)	188	oop. def. (2nim)
Q2	261 (1g)	266	γ(nitroimidazole)
Q3	466 (all)	457	β(acetamide)
Q4	594 (all)	563	γ(acetamide)
Q5	722 (all)	719	$\beta(CF_3)$
Q6	789 (all)	795	$\gamma(CH)_{imidazole}$ (ip.)
Q7	857 (all)	845	$\delta(NO_2)+\delta(CN_2)_{imidazole}$
Q8	989 (all)	1006	$\omega(CF_2)$
Q9	1067 (all)	1084	$\beta(CH)_{imidazole}$
Q10	1134 (all)	1147	$\nu_{as}(CF_2)$
Q11	1147 (all)	1158	$\nu_{sym}(CF_2)$
Q12	1244 (all)	1254	ν(C-N)
Q13	1260 (all)	1270	$\beta(CH)+\nu_{sym}(NO_2)$+ip. def.(imidazole)
Q14	1280 (all)	1299	ν(CC)
Q15	1318 (all)	1323	$\nu(CN)_{imidazole} + \delta(NO_2)$
Q16	1425 (all)	1435	$\beta(CH_2)$
Q17	1459 (all)	1475	$\nu(CN)_{imidazole}+\beta(CH_2)+\beta(CH)$
Q18	1517 (all)	1535	β(NH)
Q19	1539 (all)	1558	$\nu_{asym}(NO_2)$
Q20	1705 (all)	1732	ν(C=O)
Q21	2968 (all)	2958	$\nu_{sym}(CH_2)$
Q22	3005 (all)	2989	$\nu_{sym}(CH_2)$
Q23	3029 (all)	2994	$\nu_{asym}(CH_2)$
Q24	3140 (all)	3122	ν_{asym}(CH)
Q25	3418 (2g)	3368	ν(NH)
Q26	3444 (1g)		ν(NH)

[a] oop. – out-of-plane; ip. – in plane; def. – deformation; ρ - rocking; ν - stretching; β - in plane bending XYH angles; γ - out-of-plane bending; ω - wagging; δ - in-plane bending; sym. – symmetric; asym – antisymmetric.

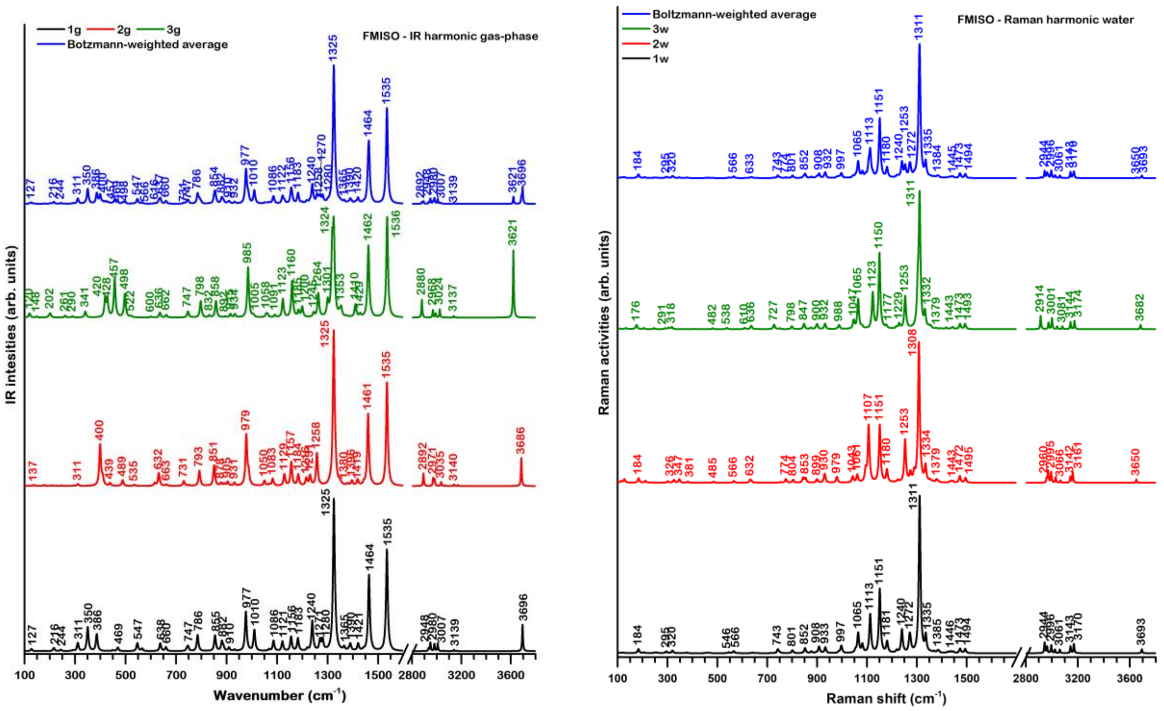

(**a**) (**b**)

Figure S2. Computed harmonic IR (a) and Raman (b) spectra of the most stable conformers of FMISO at 298K.

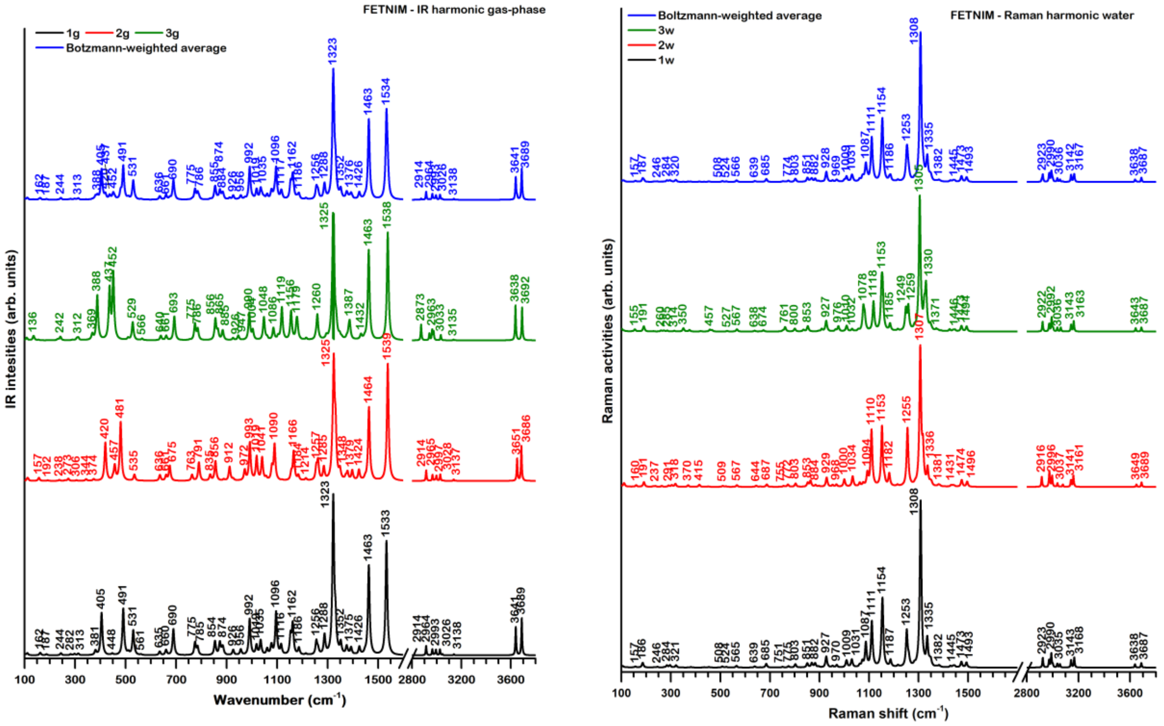

(**a**) (**b**)

Figure S3. Computed harmonic IR (a) and Raman (b) spectra of the most stable conformers of FETNIM at 298K.

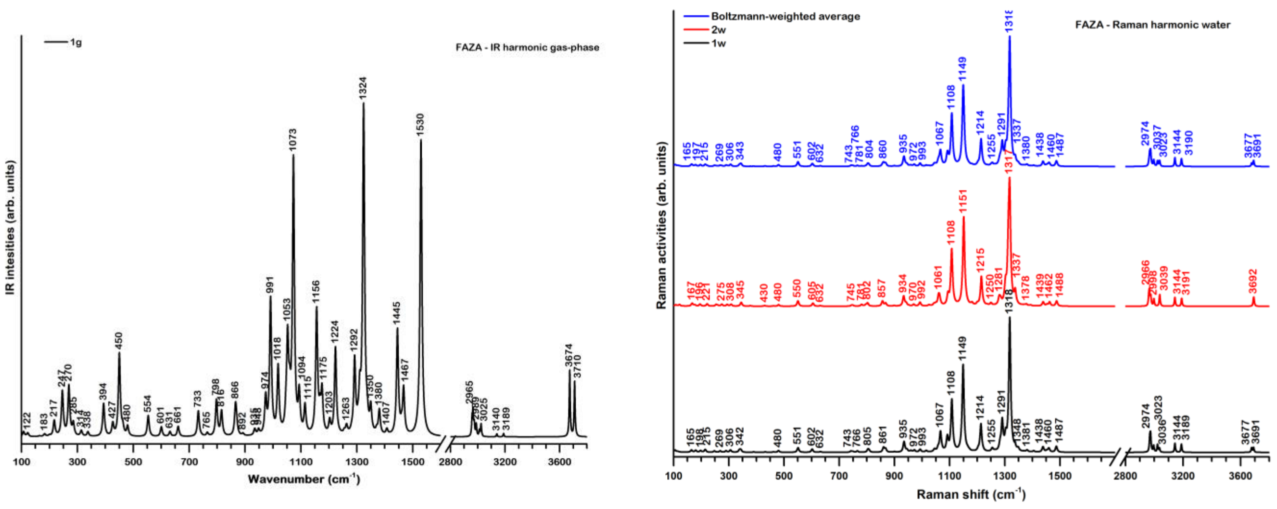

(**a**) (**b**)

Figure S4. Computed harmonic IR (a) and Raman (b) spectra of the most stable conformers of FAZA at 298K.

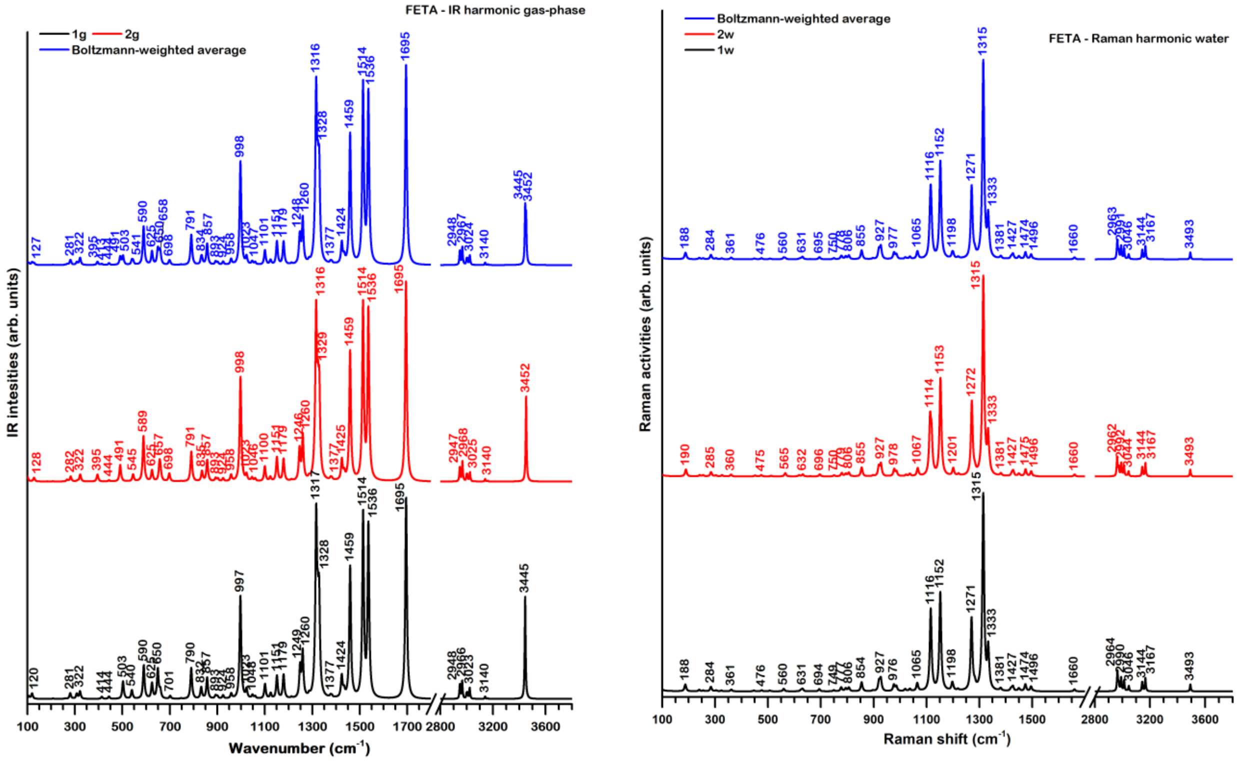

(**a**) (**b**)

Figure S5. Computed harmonic IR (a) and Raman (b) spectra of the most stable conformers of FETA at 298K.

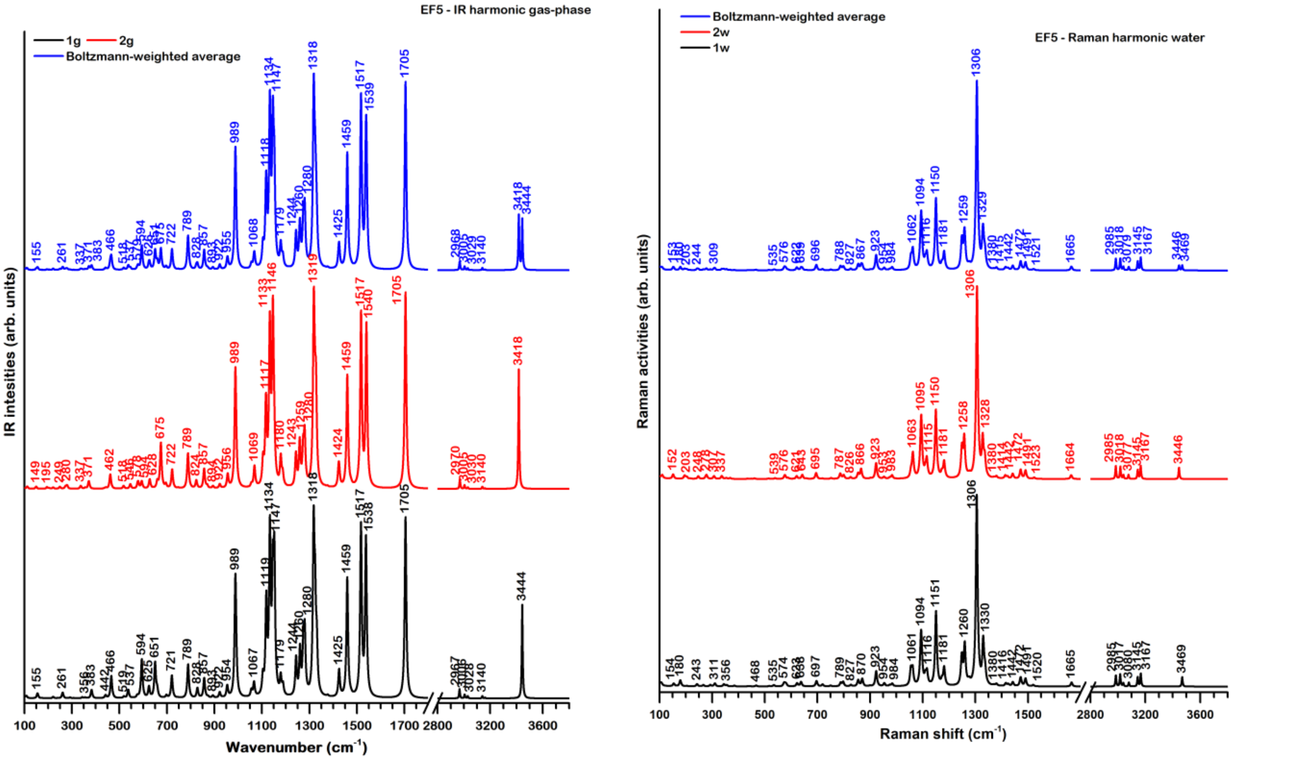

(**a**) (**b**)

Figure S6. Computed harmonic IR (a) and Raman (b) spectra of the most stable conformers of EF5 at 273K.

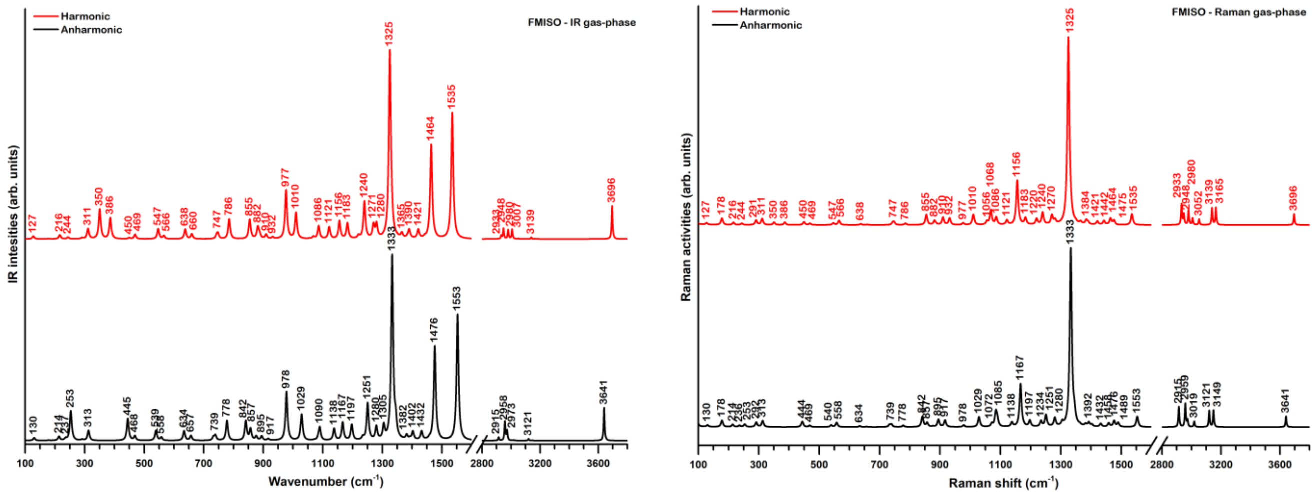

(**a**) (**b**)

Figure S7. Computed IR (a) and Raman (b) spectra for 1g FMISO in gas-phase in the harmonic (red) and anharmonic (black) approximation.

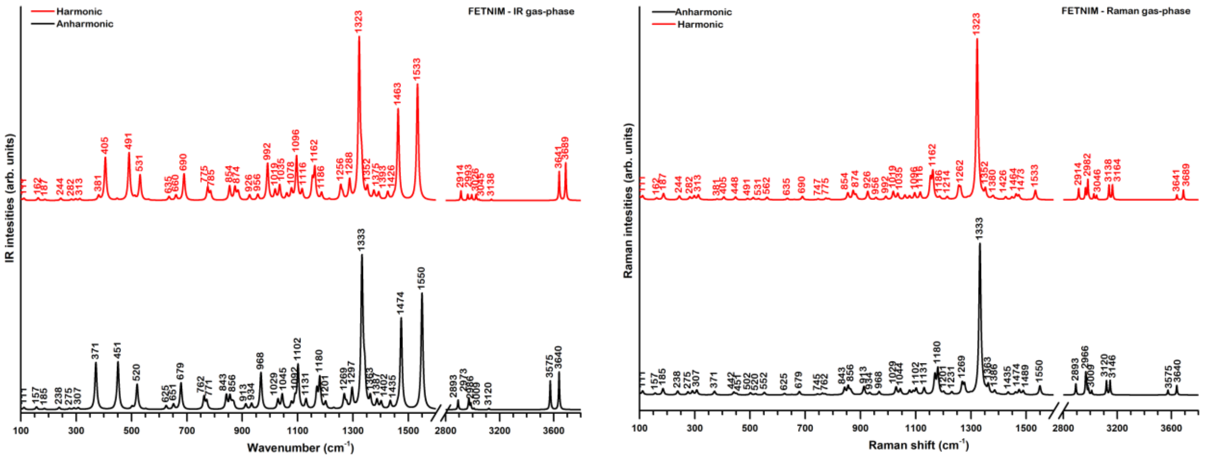

(**a**) (**b**)

Figure S8. Computed IR (a) and Raman (b) spectra for 1g FETNIM in gas-phase in the harmonic (red) and anharmonic (black) approximation.

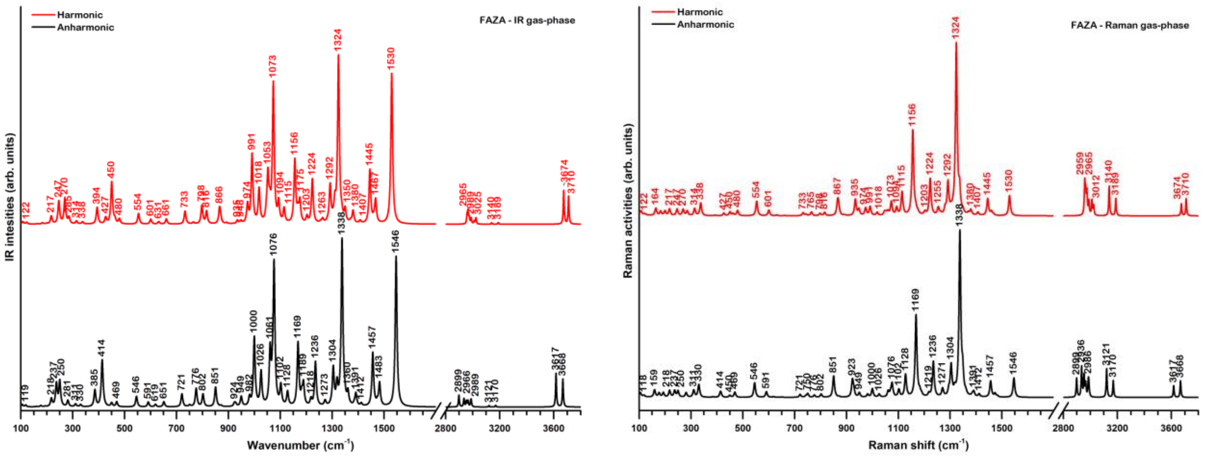

(**a**) (**b**)

Figure S9. Computed IR (a) and Raman (b) spectra for 1g FAZA in gas-phase in the harmonic (red) and anharmonic (black) approximation.

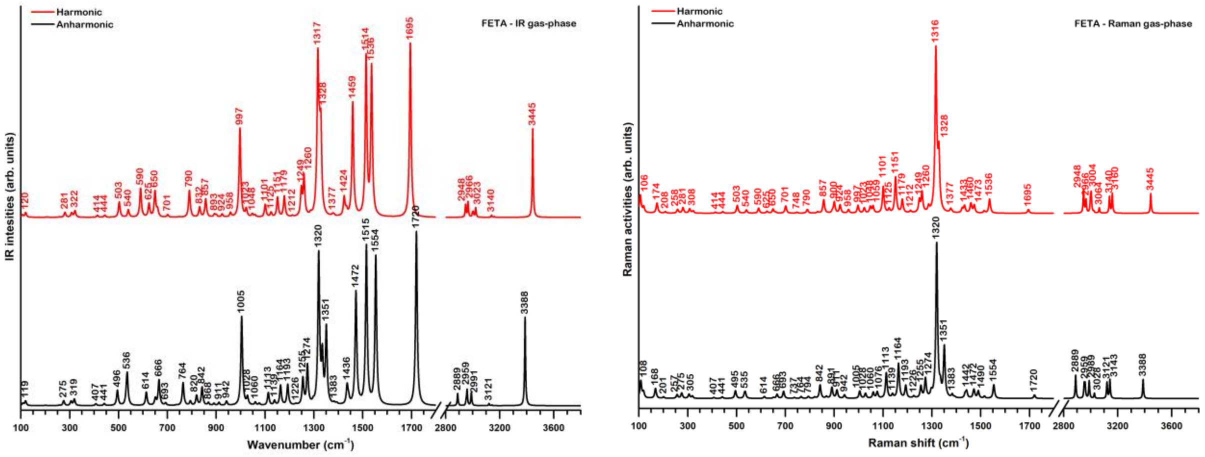

(**a**) (**b**)

Figure S10. Computed IR (a) and Raman (b) spectra for 1g FETA in gas-phase in the harmonic (red) and anharmonic (black) approximation.

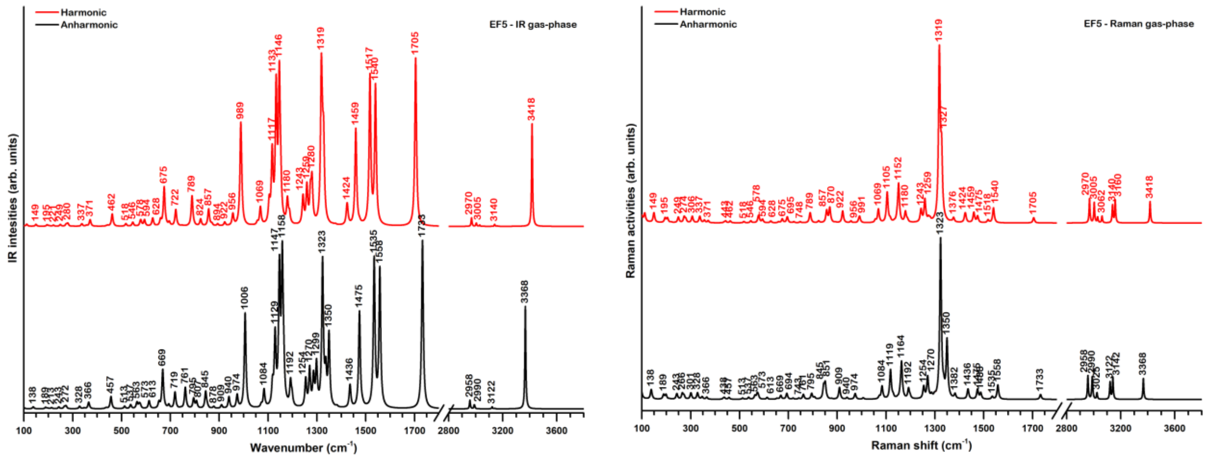

(**a**) (**b**)

Figure S11. Computed IR (a) and Raman (b) spectra for 1g EF5 in gas-phase in the harmonic (red) and anharmonic (black) approximation.

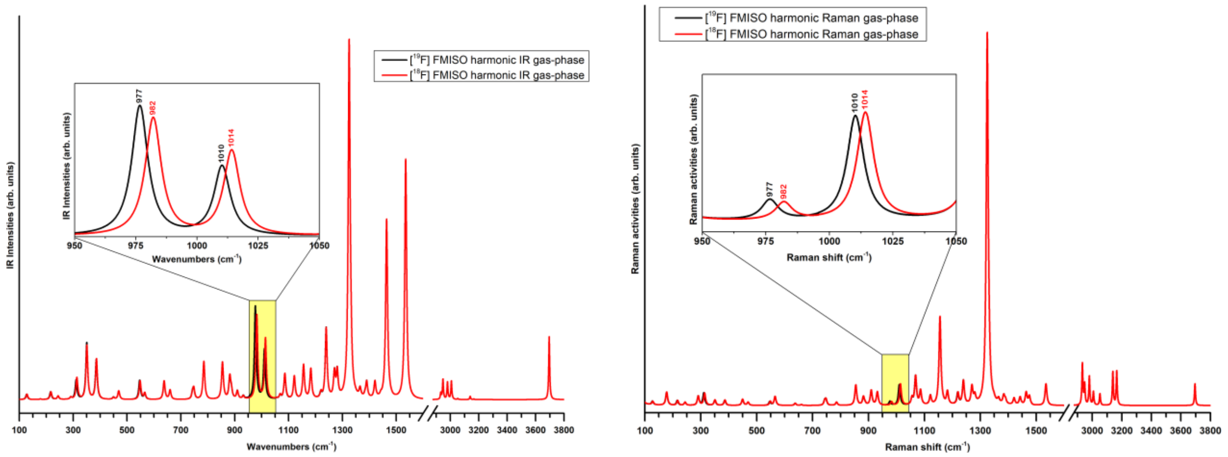

(**a**) (**b**)

Figure S12. Comparison of the computed IR (a) and Raman (b) harmonic spectra of 1g [^{19}F]FMISO (black) and [^{18}F]FMISO (red) in gas-phase in the harmonic approximation.

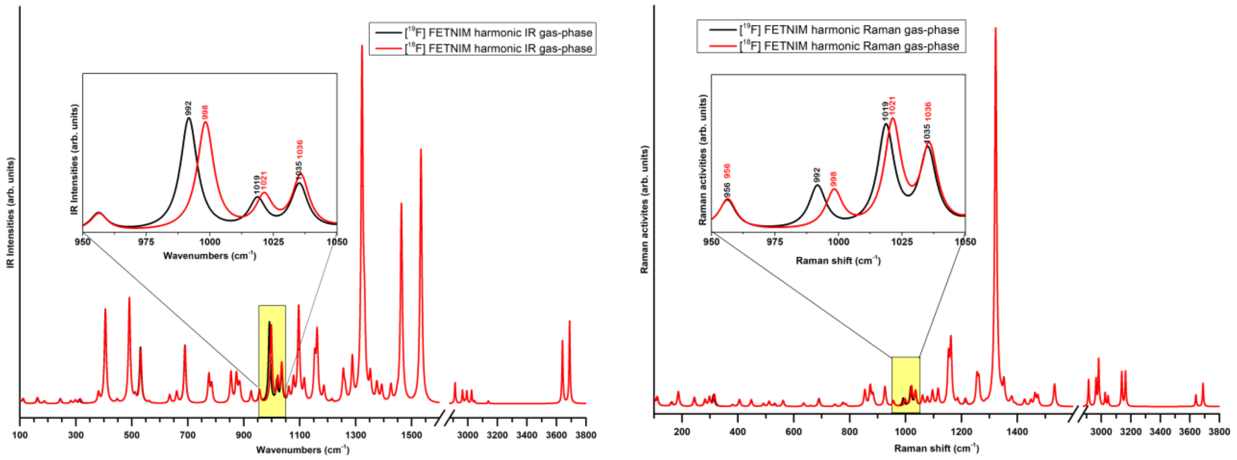

(**a**) (**b**)

Figure S13. Comparison of the computed IR (a) and Raman (b) harmonic spectra of 1g [^{19}F]FETNIM (black) and [^{18}F]FETNIM (red) in gas-phase in the harmonic approximation.

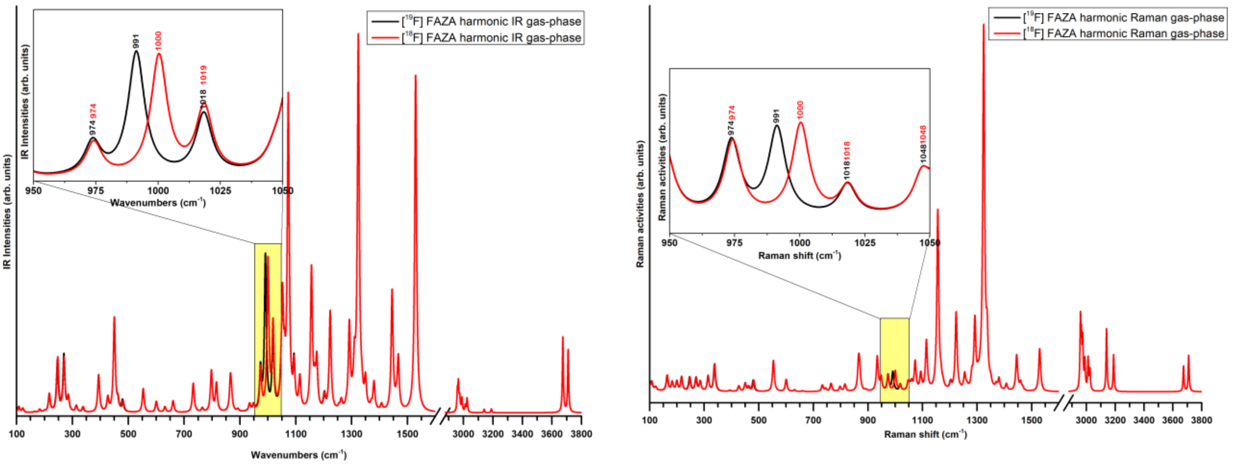

(**a**) (**b**)

Figure S14. Comparison of the computed IR (a) and Raman (b) harmonic spectra of 1g [19F]FAZA (black) and [18F]FAZA (red) in gas-phase in the harmonic approximation.

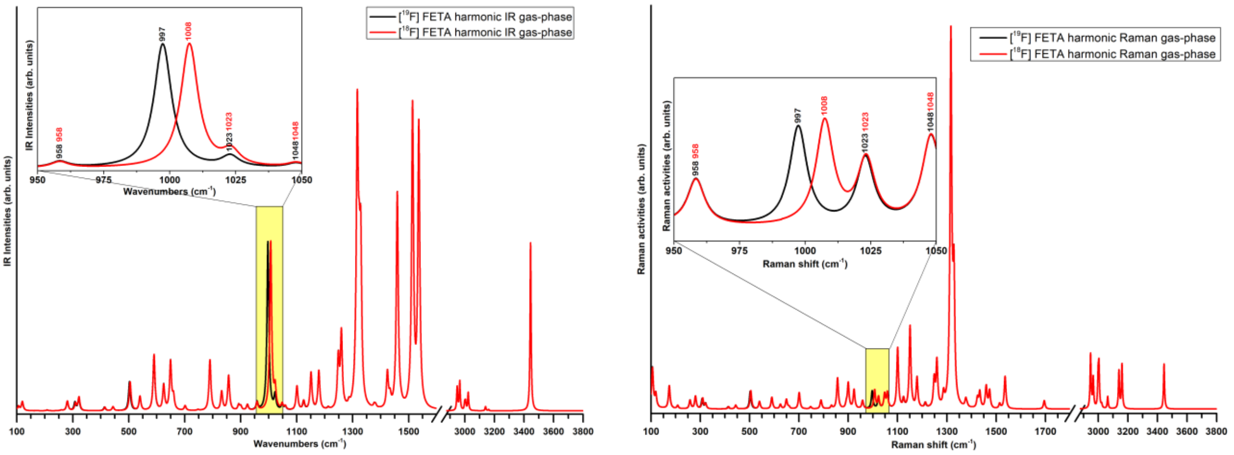

(**a**) (**b**)

Figure S15. Comparison of the computed IR (a) and Raman (b) harmonic spectra of 1g [19F]FETA (black) and [18F]FETA (red) in gas-phase in the harmonic approximation.

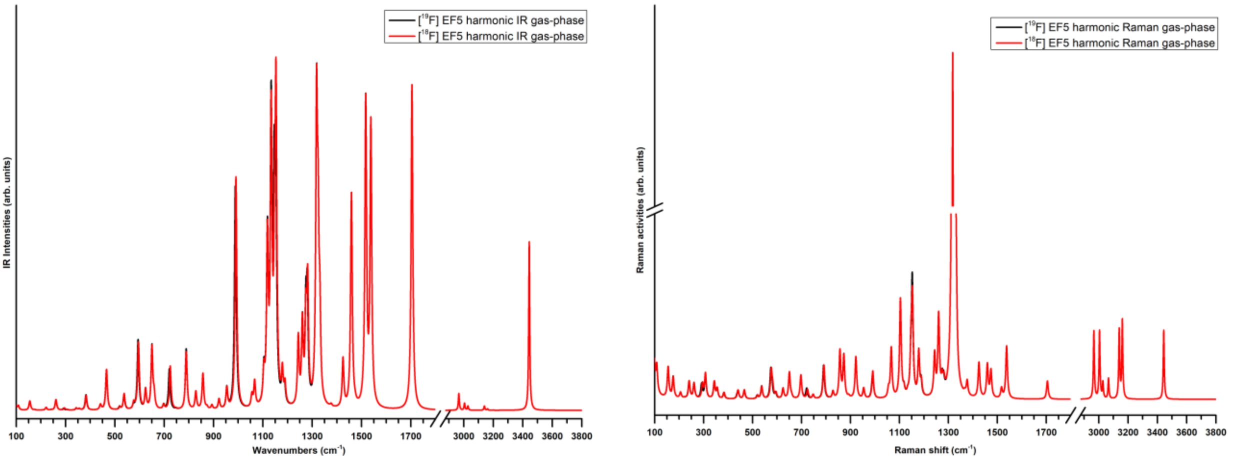

(**a**) (**b**)

Figure S16. Comparison of the computed IR (a) and Raman (b) harmonic spectra of 1g [^{19}F]EF5 (black) and [^{18}F]EF5 (red) in gas-phase in the harmonic approximation.

Table S6. Calculated NMR data for FETNIM

Nucleus	Calculated data	
	Chemical shift (ppm)	J coupling (Hz)
H(14) H(15)	7.50, 7.39	$^3J_{H14H15}$=0.77
H(16)	4.37	$^2J_{H16H17}$=12.75
H(17)	4.94	$^2J_{H17H16}$ = 12.75<br>$^4J_{H17H19}$=2.32
H(18)	3.96	-
H(20)	2.04	-
H(22)	2.38	-
H(23)	4.37	$3J_{H23H24}$ = 8.57
H(24)	4.83	
H(25)	4.88	
C(2)	154.64	-
C(4) C(5)	139.25 136.19	
C(9)	60.25	
C(10)	74.24	$^3J_{C10F13}$ = 15.15
C(11)	76.54	$^1J_{C11H21}$ = 147.9
C(12)	94.08	
F(13)	-269.62[a], -176.69[b]	$^1J_{F13C12}$ = -191.93<br>$^2J_{F13C11}$ = 13.78

Solvent: [a] $CFCl_3$; [b] CF_3COOH.

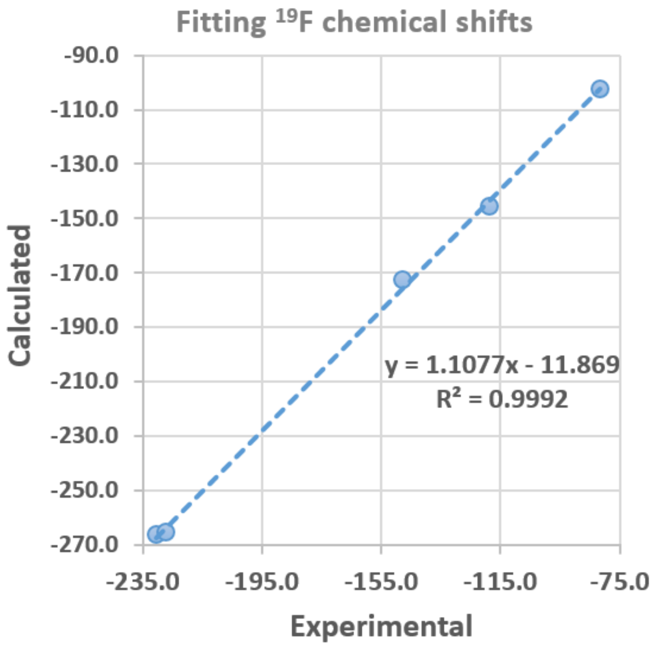

Figure S17. Least-squares linear fit of calculated vs experimental values for the chemical shifts of ^{19}F nuclei. The experimental and calculated data used for this fit are those reported in Tables 4, 5 and 7 for F nuclei.

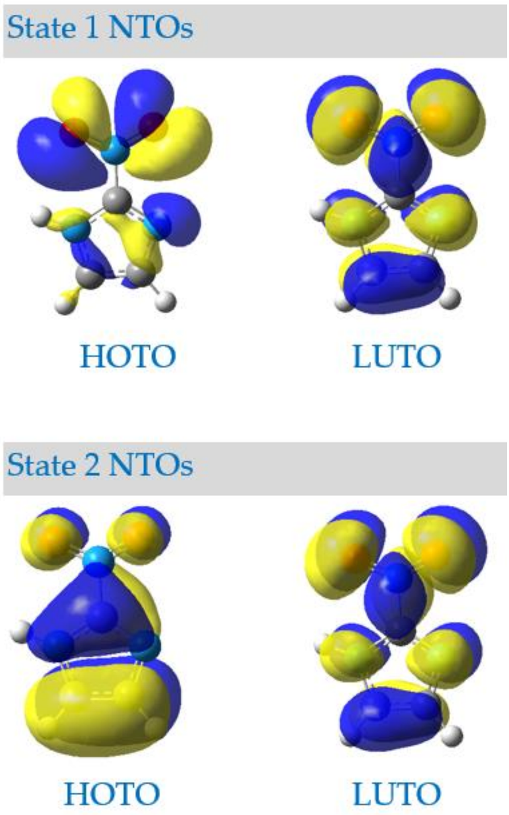

Figure S18. Natural transition orbitals corresponding to the first two excited singlet states of **2nim**

Table S7. Cartesian coordinates for the B3LYP/6-311+G(d,p) optimized structures of **2nim**.

Atom list	Cartesian coordinates					
	Gas-phase			Water		
	X	Y	Z	X	Y	Z
N	0.75438000	-1.18545000	0.00002700	-0.74417000	-1.18069100	-0.00000900
C	0.02354800	-0.10186200	-0.00000400	-0.02002900	-0.08328700	-0.00000100
N	0.75924900	1.04278600	-0.00003600	-0.77139600	1.04890600	0.00000900
C	2.06962300	0.65185000	-0.00001200	-2.07070000	0.64601900	0.00000600
C	2.04227500	-0.72962700	0.00001900	-2.03326000	-0.73980300	-0.00000500
N	-1.42037100	-0.02970700	0.00000000	1.40920900	-0.02320800	-0.00000100
O	-1.89514200	1.11133700	0.00004500	1.91772500	1.10254400	0.00000800
O	-2.05485300	-1.06662100	-0.00003900	2.04026600	-1.07427700	-0.00000900
H	0.37345800	1.97643100	-0.00000300	-0.41919100	1.99750500	0.00001700
H	2.88166700	1.35972900	-0.00004000	-2.88875200	1.34656200	0.00001100
H	2.87935000	-1.40945900	0.00003700	-2.86754900	-1.42282600	-0.00001100

Table S8. Cartesian coordinates for the B3LYP/6-311+G(d,p) optimized structures of FMISO for the most stable conformers in gas-phase and water (1-3g and 1-3w).

Atom list	Cartesian coordinates					
	1g			2g		
	X	Y	Z	X	Y	Z
N	2.70300000	0.69500000	-0.40300000	-2.28400000	0.97200000	-0.72000000
C	1.69200000	-0.04100000	-0.00900000	-1.52100000	0.14300000	-0.05100000
N	0.62600000	0.70200000	0.44600000	-0.39200000	0.74100000	0.46700000
C	1.02200000	2.00600000	0.30900000	-0.48200000	2.05300000	0.07900000
C	2.29900000	1.97900000	-0.21300000	-1.64600000	2.17100000	-0.65100000
N	1.70000000	-1.48600000	-0.06300000	-1.83400000	-1.26100000	0.09400000
O	0.63100000	-2.06300000	0.17600000	-0.92800000	-2.00200000	0.50000000
O	2.74500000	-2.04000000	-0.34300000	-2.95600000	-1.62800000	-0.19800000
C	-0.67800000	0.29100000	0.98300000	0.67500000	0.21900000	1.33700000
C	-1.72800000	0.10700000	-0.11000000	2.00300000	-0.02100000	0.61800000
C	-2.97200000	-0.56700000	0.44900000	1.91300000	-0.98800000	-0.55200000
O	-2.03100000	1.40300000	-0.61400000	2.49300000	1.24700000	0.19200000
F	-3.92700000	-0.63400000	-0.57800000	3.20100000	-1.07500000	-1.12000000
H	0.36700000	2.81800000	0.57900000	0.28500000	2.76100000	0.34400000
H	2.93400000	2.81400000	-0.46000000	-2.04500000	3.05200000	-1.12900000
H	-1.00400000	1.08400000	1.65800000	0.32800000	-0.70100000	1.79700000
H	-0.54600000	-0.62900000	1.54600000	0.84700000	0.96400000	2.11500000
H	-1.31800000	-0.52900000	-0.90300000	2.68600000	-0.45200000	1.36500000
H	-2.68200000	1.31000000	-1.31900000	3.27500000	1.10000000	-0.35300000
H	-2.76200000	-1.58800000	0.77400000	1.61500000	-1.98800000	-0.23600000
H	-3.41500000	0.01400000	1.26200000	1.24700000	-0.62100000	-1.33400000
	3g			1w		
N	1.92400000	1.53200000	-0.46100000	2.70500000	0.64200000	-0.40200000
C	1.39000000	0.43200000	0.01500000	1.66700000	-0.06500000	-0.00400000
N	0.10500000	0.60700000	0.47700000	0.63300000	0.71400000	0.46200000
C	-0.16600000	1.93200000	0.25800000	1.07300000	1.99900000	0.33400000
C	0.96100000	2.47900000	-0.32100000	2.34800000	1.93500000	-0.19900000
N	2.11500000	-0.81700000	0.06700000	1.64300000	-1.49600000	-0.05000000
O	1.48900000	-1.83100000	0.42400000	0.59600000	-2.06900000	0.27100000
O	3.29100000	-0.80700000	-0.23100000	2.66100000	-2.07800000	-0.41100000
C	-0.84900000	-0.33100000	1.09100000	-0.68900000	0.35300000	0.99900000
C	-1.74200000	-1.07700000	0.08400000	-1.71600000	0.10600000	-0.10600000
C	-2.66200000	-0.15200000	-0.70600000	-3.01000000	-0.42200000	0.49300000
O	-1.02900000	-1.79900000	-0.89900000	-1.92500000	1.34600000	-0.77800000
F	-3.42000000	0.62800000	0.18300000	-3.92500000	-0.63900000	-0.56100000
H	-1.11600000	2.35900000	0.53500000	0.46100000	2.83400000	0.62900000
H	1.11900000	3.49800000	-0.63600000	3.00700000	2.75400000	-0.43900000
H	-1.48700000	0.25300000	1.75500000	-1.00900000	1.19300000	1.61600000

H	-0.29000000	-1.04500000	1.69100000	-0.58300000	-0.52500000	1.62800000
H	-2.36700000	-1.74900000	0.69300000	-1.32700000	-0.64200000	-0.80500000
H	-0.24100000	-2.19400000	-0.50100000	-2.44800000	1.18000000	-1.57100000
H	-2.08800000	0.52300000	-1.34300000	-2.86100000	-1.37900000	0.99600000
H	-3.35100000	-0.73600000	-1.31700000	-3.46900000	0.30200000	1.16900000
	**2w**			**3w**		
N	-2.71000000	-0.48500000	0.22300000	-2.30600000	0.92000000	-0.71800000
C	-1.55300000	0.07200000	-0.07800000	-1.50800000	0.11400000	-0.04600000
N	-0.57500000	-0.84800000	-0.37000000	-0.40900000	0.75800000	0.47500000
C	-1.17900000	-2.06300000	-0.23000000	-0.55000000	2.06100000	0.09200000
C	-2.49100000	-1.82000000	0.13600000	-1.71800000	2.14000000	-0.64400000
N	-1.38100000	1.49500000	-0.11100000	-1.78400000	-1.28500000	0.10300000
O	-0.24500000	1.94700000	-0.31100000	-0.90900000	-2.00100000	0.60300000
O	-2.37000000	2.19600000	0.05900000	-2.87300000	-1.70000000	-0.28000000
C	0.84100000	-0.68200000	-0.74000000	0.69500000	0.28400000	1.33500000
C	1.76900000	-0.68100000	0.48800000	2.01200000	0.05400000	0.59100000
C	3.21900000	-0.50300000	0.06700000	1.92600000	-0.99400000	-0.50600000
O	1.38100000	0.27700000	1.46400000	2.43800000	1.30900000	0.06500000
F	3.37300000	0.74000000	-0.59100000	3.21500000	-1.11500000	-1.08200000
H	-0.64500000	-2.98200000	-0.40700000	0.17500000	2.80600000	0.37100000
H	-3.26800000	-2.54100000	0.33300000	-2.14700000	3.01000000	-1.11400000
H	0.94300000	0.23900000	-1.30600000	0.38200000	-0.62500000	1.83600000
H	1.09800000	-1.51800000	-1.39000000	0.85900000	1.05800000	2.08400000
H	1.70300000	-1.65300000	0.98700000	2.72600000	-0.29700000	1.34600000
H	1.31100000	1.14200000	1.03800000	3.26500000	1.17300000	-0.41300000
H	3.86800000	-0.48600000	0.94200000	1.65000000	-1.97200000	-0.11200000
H	3.53900000	-1.28200000	-0.62800000	1.25300000	-0.69700000	-1.31100000

Table S9. Cartesian coordinates for the B3LYP/6-311+G(d,p) optimized structures of FETNIM for the most stable conformers in gas-phase and water (1-3g and 1-3w).

Atom list	Cartesian coordinates					
	1g			2g		
	X	Y	Z	X	Y	Z
N	3.40700000	0.44400000	-0.38800000	2.67200000	1.36600000	0.50800000
C	2.31200000	-0.15500000	0.01500000	2.06600000	0.27300000	0.11200000
N	1.31500000	0.72300000	0.38000000	0.97300000	0.51600000	-0.68900000
C	1.85100000	1.96400000	0.17200000	0.91200000	1.88200000	-0.78800000
C	3.13400000	1.77200000	-0.29900000	1.96000000	2.38300000	-0.04500000
N	2.17700000	-1.59200000	0.06900000	2.51300000	-1.05300000	0.48300000
O	1.05400000	-2.04300000	0.33500000	1.78700000	-1.99900000	0.15800000

O	3.16400000	-2.26800000	-0.15300000	3.56300000	-1.15000000	1.08900000
C	-0.04500000	0.48500000	0.88200000	0.03200000	-0.39100000	-1.36600000
C	-1.04600000	0.21400000	-0.25400000	-1.09400000	-0.93900000	-0.46400000
C	-2.41600000	-0.17000000	0.32100000	-1.90100000	0.16600000	0.22400000
C	-3.35400000	-0.68500000	-0.75800000	-2.93800000	-0.40400000	1.17700000
F	-4.62100000	-0.87800000	-0.17200000	-3.70400000	0.67100000	1.67300000
H	1.28400000	2.85800000	0.37000000	0.13900000	2.36200000	-1.36700000
H	3.86000000	2.51900000	-0.57600000	2.23100000	3.41500000	0.11300000
H	-0.34300000	1.37900000	1.42900000	-0.41000000	0.18100000	-2.18200000
H	-0.01900000	-0.35600000	1.57000000	0.57800000	-1.23600000	-1.77800000
H	-0.66800000	-0.61300000	-0.85700000	-0.65000000	-1.59000000	0.29100000
O	-1.15800000	1.33300000	-1.12200000	-1.93400000	-1.76300000	-1.25800000
H	-1.74900000	1.96600000	-0.69100000	-2.48300000	-1.18000000	-1.79900000
O	-2.93900000	1.01500000	0.92600000	-2.52800000	0.92000000	-0.81800000
H	-3.86500000	0.86200000	1.14700000	-3.19500000	1.49100000	-0.41900000
H	-2.28800000	-0.95400000	1.07900000	-1.22500000	0.81400000	0.79700000
H	-3.47600000	0.03600000	-1.56700000	-3.62300000	-1.08500000	0.67300000
H	-3.02000000	-1.64800000	-1.14900000	-2.46800000	-0.89700000	2.03000000
	**3g**			**1w**		
N	-3.35000000	-0.61100000	0.38200000	3.38900000	-0.49300000	0.38800000
C	-2.26600000	0.01400000	-0.01100000	2.30500000	0.13800000	-0.02000000
N	-1.27100000	-0.83800000	-0.43100000	1.29400000	-0.71500000	-0.39300000
C	-1.79600000	-2.09300000	-0.26800000	1.79700000	-1.96600000	-0.19100000
C	-3.07200000	-1.93100000	0.23100000	3.08500000	-1.81000000	0.28900000
N	-2.16800000	1.45900000	-0.01800000	2.22100000	1.56600000	-0.07500000
O	-1.09500000	1.95900000	-0.38500000	1.14200000	2.07200000	-0.40100000
O	-3.14400000	2.09300000	0.32900000	3.22500000	2.21400000	0.20800000
C	0.09900000	-0.58700000	-0.90300000	-0.06600000	-0.45200000	-0.89000000
C	1.11500000	-0.53400000	0.25200000	-1.05300000	-0.14500000	0.25000000
C	2.53300000	-0.34000000	-0.31000000	-2.44400000	0.15500000	-0.32600000
C	3.56600000	-0.28500000	0.80200000	-3.38700000	0.68000000	0.74200000
F	4.82800000	-0.07900000	0.20800000	-4.65700000	0.87700000	0.14700000
H	-1.23100000	-2.97500000	-0.52500000	1.21600000	-2.84800000	-0.40700000
H	-3.79300000	-2.69400000	0.47800000	3.78800000	-2.58200000	0.56000000
H	0.11700000	0.34100000	-1.46500000	-0.38000000	-1.34800000	-1.42300000
H	0.35400000	-1.40600000	-1.57900000	-0.02800000	0.37800000	-1.59100000
H	1.09300000	-1.48900000	0.78700000	-0.70000000	0.73100000	0.79700000
O	0.78300000	0.44300000	1.21800000	-1.10700000	-1.20900000	1.19300000
H	0.63700000	1.28600000	0.76600000	-1.63100000	-1.92200000	0.79900000
O	2.53200000	0.86600000	-1.06500000	-2.92300000	-1.07500000	-0.87700000
H	3.44300000	1.09100000	-1.28400000	-3.83700000	-0.95400000	-1.16200000

H	2.77400000	-1.19300000	-0.96200000	-2.35300000	0.91100000	-1.11500000
H	3.38100000	0.54900000	1.47900000	-3.52400000	-0.03400000	1.55300000
H	3.61400000	-1.22500000	1.35700000	-3.05500000	1.64500000	1.12800000
	**2w**			**3w**		
N	3.32500000	-0.63100000	-0.40500000	2.70500000	1.36100000	0.50600000
C	2.24900000	0.00800000	0.01200000	2.07700000	0.27200000	0.11200000
N	1.26700000	-0.83800000	0.46900000	0.98300000	0.53600000	-0.67800000
C	1.77900000	-2.09300000	0.30900000	0.94000000	1.89800000	-0.77500000
C	3.04500000	-1.94600000	-0.22700000	2.00400000	2.38700000	-0.03800000
N	2.17100000	1.44100000	0.01600000	2.51900000	-1.04500000	0.46800000
O	1.16800000	1.97600000	0.49900000	1.82900000	-2.00400000	0.10800000
O	3.11500000	2.06400000	-0.45900000	3.55800000	-1.14900000	1.11100000
C	-0.10000000	-0.57600000	0.94700000	0.02200000	-0.36100000	-1.34500000
C	-1.11300000	-0.54100000	-0.21300000	-1.07900000	-0.90800000	-0.41400000
C	-2.52200000	-0.26600000	0.33100000	-1.96400000	0.19400000	0.18000000
C	-3.57400000	-0.41900000	-0.75400000	-2.95600000	-0.36900000	1.18200000
F	-4.83700000	-0.11700000	-0.18700000	-3.74800000	0.70300000	1.66500000
H	1.21500000	-2.96900000	0.58500000	0.17900000	2.39600000	-1.35200000
H	3.75200000	-2.72100000	-0.47900000	2.28500000	3.41600000	0.11500000
H	-0.10600000	0.35900000	1.49600000	-0.42900000	0.21600000	-2.15100000
H	-0.35400000	-1.38700000	1.63000000	0.56200000	-1.20200000	-1.77100000
H	-1.11200000	-1.51400000	-0.70900000	-0.61400000	-1.47100000	0.39600000
O	-0.74700000	0.40000000	-1.21300000	-1.85700000	-1.84300000	-1.15500000
H	-0.83400000	1.28500000	-0.83200000	-2.44200000	-1.34100000	-1.73900000
O	-2.50800000	1.06400000	0.85100000	-2.63400000	0.81300000	-0.92000000
H	-3.40100000	1.29600000	1.13300000	-3.26500000	1.45700000	-0.57900000
H	-2.74200000	-0.98200000	1.13300000	-1.33300000	0.92700000	0.69800000
H	-3.41700000	0.28200000	-1.57300000	-3.63800000	-1.08500000	0.72500000
H	-3.62000000	-1.44400000	-1.12400000	-2.44900000	-0.81100000	2.04100000

Table S10. Cartesian coordinates for the B3LYP/6-311+G(d,p) optimized structures of FETA for the most stable conformers in gas-phase and water (1-2g and 1-2w).

Atom list	Cartesian coordinates					
	1g			2g		
	X	Y	Z	X	Y	Z
N	3.20900000	-0.12500000	-0.89800000	2.79200000	-0.00100000	1.26000000
C	2.22500000	0.25200000	-0.11700000	2.02000000	0.24700000	0.22900000
N	1.48800000	-0.80500000	0.37200000	1.39300000	-0.88000000	-0.25900000
C	2.07400000	-1.92000000	-0.16500000	1.81700000	-1.89800000	0.55300000
C	3.12500000	-1.48000000	-0.94300000	2.67300000	-1.33500000	1.47900000

N	1.95000000	1.63600000	0.17500000	1.85000000	1.57100000	-0.31600000
O	0.86600000	1.89900000	0.72600000	0.94400000	1.73100000	-1.15400000
O	2.78300000	2.46400000	-0.13300000	2.59100000	2.45000000	0.07200000
C	0.33400000	-0.85500000	1.28300000	0.46700000	-1.07300000	-1.38500000
C	-0.99700000	-0.95500000	0.50900000	-1.00200000	-1.05300000	-0.91600000
N	-1.49500000	0.22600000	0.06700000	-1.58000000	0.17300000	-0.88400000
C	-2.70400000	0.30400000	-0.74400000	-2.95800000	0.37200000	-0.45400000
C	-3.95300000	0.40400000	0.12700000	-3.05900000	0.53400000	1.06100000
H	1.69400000	-2.90600000	0.05000000	1.47500000	-2.90900000	0.40200000
H	3.81400000	-2.07100000	-1.52600000	3.19900000	-1.82700000	2.28200000
F	-5.07200000	0.52000000	-0.70600000	-4.39900000	0.75900000	1.39800000
O	-1.52200000	-2.04000000	0.32200000	-1.56400000	-2.09100000	-0.60700000
H	0.36300000	0.01200000	1.93600000	0.65500000	-2.06500000	-1.79200000
H	0.42600000	-1.76500000	1.87300000	0.67100000	-0.32100000	-2.14200000
H	-0.95700000	1.06400000	0.24800000	-1.00000000	0.97600000	-1.09500000
H	-2.63500000	1.18000000	-1.39100000	-3.35200000	1.26200000	-0.94700000
H	-2.76900000	-0.58800000	-1.37000000	-3.54800000	-0.49000000	-0.76700000
H	-3.92300000	1.29000000	0.76800000	-2.48100000	1.39200000	1.41300000
H	-4.07900000	-0.49200000	0.73700000	-2.73100000	-0.37200000	1.57600000
		**1w**			**2w**	
N	3.34500000	0.24700000	0.65200000	3.39700000	-0.49300000	-0.64200000
C	2.19700000	0.38100000	0.02200000	2.35600000	0.07500000	-0.06900000
N	1.64800000	-0.82000000	-0.35500000	1.48000000	-0.83300000	0.47300000
C	2.52400000	-1.76700000	0.09000000	2.02100000	-2.05600000	0.20100000
C	3.56300000	-1.09100000	0.70400000	3.20100000	-1.82700000	-0.48200000
N	1.59700000	1.65300000	-0.24300000	2.17300000	1.49400000	-0.01500000
O	0.51100000	1.66600000	-0.83400000	1.15300000	1.92100000	0.53500000
O	2.18900000	2.66100000	0.12900000	3.03400000	2.20700000	-0.51800000
C	0.38700000	-1.11700000	-1.02700000	0.20600000	-0.63100000	1.15500000
C	-0.81600000	-0.90100000	-0.09300000	-0.90300000	-0.20600000	0.17700000
N	-2.00800000	-0.90200000	-0.73100000	-2.01900000	0.25100000	0.78700000
C	-3.28000000	-0.79100000	-0.02900000	-3.21200000	0.66100000	0.05800000
C	-3.72700000	0.66300000	0.07100000	-4.20600000	-0.49000000	-0.06000000
H	2.34500000	-2.81800000	-0.06500000	1.53800000	-2.96800000	0.51200000
H	4.44100000	-1.51100000	1.16900000	3.90200000	-2.55600000	-0.85400000
F	-4.96500000	0.70600000	0.74900000	-5.33900000	-0.03300000	-0.76600000
O	-0.68300000	-0.78500000	1.11800000	-0.77200000	-0.30900000	-1.03600000
H	0.42000000	-2.16700000	-1.32200000	0.32500000	0.10300000	1.94900000
H	0.29700000	-0.51100000	-1.92500000	-0.07000000	-1.58400000	1.60600000
H	-2.02600000	-0.97400000	-1.73800000	-2.03500000	0.31300000	1.79500000
H	-4.02900000	-1.37400000	-0.56800000	-3.67300000	1.49800000	0.58300000

H	-3.16400000	-1.21200000	0.97000000	-2.91300000	0.99500000	-0.93600000
H	-3.88000000	1.10800000	-0.91400000	-4.54900000	-0.83100000	0.91900000
H	-3.01900000	1.26300000	0.64500000	-3.78800000	-1.32700000	-0.62100000

Table S11. Cartesian coordinates for the B3LYP/6-311+G(d,p) optimized structures of FAZA for the most stable conformers in gas-phase and water (1-2g and 1-2w).

Atom list	Cartesian coordinates					
	1g			2g		
	X	Y	Z	X	Y	Z
N	-3.22400000	1.22300000	0.05200000	3.53600000	-0.26300000	-0.12600000
C	-2.38300000	0.23100000	-0.12400000	2.24800000	-0.41700000	0.04100000
N	-1.08800000	0.65100000	-0.34100000	1.50700000	0.64700000	-0.42300000
C	-1.14400000	2.01400000	-0.29800000	2.43900000	1.52400000	-0.92700000
C	-2.46500000	2.34300000	-0.05400000	3.67400000	0.95500000	-0.72200000
N	-2.77200000	-1.15400000	-0.08600000	1.69100000	-1.58600000	0.69200000
O	-1.86500000	-1.99800000	-0.19600000	0.58300000	-1.46100000	1.21900000
O	-3.95000000	-1.42000000	0.05100000	2.35900000	-2.60300000	0.69400000
C	0.12900000	-0.15700000	-0.60900000	0.06100000	0.87900000	-0.56500000
C	0.67700000	-0.87000000	0.65700000	-0.70400000	1.24300000	0.71300000
C	1.87700000	-0.00400000	1.06100000	-2.10100000	0.70500000	0.39700000
C	2.32300000	0.62500000	-0.26500000	-1.78100000	-0.56100000	-0.41300000
O	1.13600000	0.70600000	-1.08200000	-0.57000000	-0.24900000	-1.13700000
H	-0.26500000	2.61300000	-0.45300000	2.14000000	2.45400000	-1.38300000
H	-2.89300000	3.32800000	0.04900000	4.64300000	1.35600000	-0.97200000
O	1.13800000	-2.16900000	0.33900000	-0.62700000	2.64700000	0.89300000
O	1.38900000	0.97400000	1.97700000	-2.81500000	0.48900000	1.59600000
C	3.38000000	-0.18100000	-1.01300000	-2.84200000	-0.92900000	-1.43400000
F	4.58700000	-0.07800000	-0.30400000	-4.05900000	-1.09500000	-0.74300000
H	-0.13000000	-0.88300000	-1.37700000	-0.00400000	1.72900000	-1.25100000
H	-0.05800000	-0.88400000	1.46200000	-0.30500000	0.70500000	1.57400000
H	2.65900000	-0.62200000	1.50800000	-2.62000000	1.43700000	-0.23900000
H	2.69200000	1.64300000	-0.11500000	-1.59500000	-1.39500000	0.26900000
H	0.36300000	-2.72200000	0.18100000	-0.97800000	2.86300000	1.76300000
H	2.13500000	1.44600000	2.36000000	-3.68400000	0.13200000	1.37800000
H	3.53700000	0.23400000	-2.01100000	-2.61700000	-1.86700000	-1.94400000
H	3.11600000	-1.23800000	-1.07900000	-2.98200000	-0.12900000	-2.16500000
	**1w**			**2w**		
N	-3.16800000	1.28800000	0.06600000	-3.10300000	1.35700000	0.08600000
C	-2.36900000	0.25600000	-0.11700000	-2.35700000	0.28800000	-0.10400000
N	-1.06300000	0.62200000	-0.34900000	-1.03800000	0.58900000	-0.35900000

C	-1.06100000	1.98400000	-0.31300000	-0.97400000	1.95100000	-0.33200000
C	-2.36400000	2.37400000	-0.05200000	-2.25200000	2.40400000	-0.05200000
N	-2.82100000	-1.09800000	-0.07600000	-2.87800000	-1.04300000	-0.06100000
O	-1.97400000	-1.99400000	-0.20300000	-2.08500000	-1.97300000	-0.24400000
O	-4.01900000	-1.30600000	0.08000000	-4.07700000	-1.19100000	0.15200000
C	0.12700000	-0.23300000	-0.64000000	0.11700000	-0.30900000	-0.67600000
C	0.67700000	-0.94200000	0.62400000	0.67300000	-1.03700000	0.56200000
C	1.83000000	-0.03100000	1.05900000	1.77900000	-0.08500000	1.05400000
C	2.29800000	0.60700000	-0.25700000	2.25900000	0.58700000	-0.24400000
O	1.14900000	0.58700000	-1.13900000	1.16100000	0.47700000	-1.18600000
H	-0.16300000	2.55100000	-0.48100000	-0.05600000	2.47800000	-0.51900000
H	-2.74300000	3.37800000	0.05000000	-2.58100000	3.42600000	0.04900000
O	1.20800000	-2.21300000	0.29000000	1.20700000	-2.26300000	0.08300000
O	1.27900000	0.93000000	1.95600000	1.16900000	0.83800000	1.95000000
C	3.42800000	-0.13800000	-0.95900000	3.47200000	-0.06700000	-0.89600000
F	4.60800000	0.03000000	-0.20500000	4.60000000	0.18300000	-0.08700000
H	-0.17700000	-0.94900000	-1.40100000	-0.22000000	-1.01300000	-1.43200000
H	-0.07200000	-1.01200000	1.41400000	-0.08200000	-1.19400000	1.33200000
H	2.62200000	-0.61200000	1.53700000	2.58300000	-0.63800000	1.54500000
H	2.59000000	1.64900000	-0.10800000	2.46500000	1.64700000	-0.07900000
H	0.47200000	-2.80900000	0.10200000	1.47800000	-2.80100000	0.83600000
H	1.98900000	1.49200000	2.28700000	1.85000000	1.40000000	2.33700000
H	3.60300000	0.28400000	-1.94900000	3.66100000	0.37300000	-1.87500000
H	3.23000000	-1.20800000	-1.03200000	3.35500000	-1.14700000	-0.98100000

Table S12. Cartesian coordinates for the B3LYP/6-311+G(d,p) optimized structures of EF5 for the most stable conformers in gas-phase and water (1-2g and 1-2w).

Atom list	Cartesian coordinates					
	1g			2g		
	X	Y	Z	X	Y	Z
N	4.52700000	0.19400000	0.96600000	3.48000000	-0.73900000	1.46800000
C	3.50600000	0.41200000	0.17200000	2.95500000	-0.51400000	0.28900000
N	2.95000000	-0.74900000	-0.32300000	2.61400000	0.80600000	0.09000000
C	3.69600000	-1.75800000	0.22600000	2.96000000	1.43700000	1.25600000
C	4.65600000	-1.15700000	1.01400000	3.48500000	0.47000000	2.08800000
N	3.02400000	1.73700000	-0.13200000	2.74400000	-1.55800000	-0.68300000
O	1.93900000	1.82400000	-0.73500000	1.99900000	-1.29700000	-1.64500000
O	3.69500000	2.68500000	0.21600000	3.29800000	-2.62400000	-0.51200000
C	1.82300000	-0.97900000	-1.23700000	2.05100000	1.50700000	-1.07300000
C	0.51900000	-1.24600000	-0.46000000	0.52800000	1.70200000	-0.94400000

N	-0.20400000	-0.13300000	-0.15800000	-0.21300000	0.59500000	-1.22200000
C	-1.43800000	-0.20500000	0.59000000	-1.65100000	0.58300000	-1.07700000
C	-2.63400000	0.17200000	-0.28300000	-2.07900000	-0.25200000	0.12800000
H	3.47700000	-2.79200000	0.01200000	2.80400000	2.49600000	1.38400000
H	5.42300000	-1.63100000	1.60600000	3.85900000	0.58900000	3.09300000
O	0.20500000	-2.38000000	-0.14800000	0.06600000	2.78100000	-0.62100000
C	-3.98900000	0.12100000	0.46900000	-3.61500000	-0.31000000	0.33200000
F	-2.48200000	1.44400000	-0.76100000	-1.63900000	-1.53900000	-0.01500000
F	-2.72900000	-0.65300000	-1.35800000	-1.53000000	0.23300000	1.27400000
F	-3.96600000	0.96200000	1.51900000	-4.20400000	-0.83300000	-0.75900000
F	-4.21300000	-1.12000000	0.93300000	-4.10300000	0.92800000	0.51600000
F	-5.00000000	0.46600000	-0.33000000	-3.93700000	-1.05700000	1.38900000
H	1.73500000	-0.13400000	-1.91300000	2.49500000	2.50000000	-1.10500000
H	2.04200000	-1.88200000	-1.80300000	2.31100000	0.96000000	-1.97600000
H	0.18400000	0.77200000	-0.39800000	0.27300000	-0.25800000	-1.47500000
H	-1.41300000	0.47200000	1.44800000	-2.12500000	0.16700000	-1.96900000
H	-1.56500000	-1.22800000	0.94400000	-1.99000000	1.61000000	-0.93700000
		**1w**			**2w**	
N	4.57100000	0.12000000	0.94500000	3.59400000	-0.67400000	1.42800000
C	3.53800000	0.37800000	0.17100000	2.98700000	-0.51100000	0.27000000
N	2.96100000	-0.76000000	-0.34800000	2.67900000	0.80500000	0.00700000
C	3.69800000	-1.79300000	0.15500000	3.13400000	1.49900000	1.09200000
C	4.68200000	-1.23100000	0.94900000	3.68900000	0.57200000	1.95500000
N	3.09400000	1.71200000	-0.10000000	2.69500000	-1.60700000	-0.60200000
O	2.06600000	1.86300000	-0.77500000	1.98800000	-1.39100000	-1.59900000
O	3.75500000	2.64000000	0.34800000	3.15800000	-2.70500000	-0.32400000
C	1.80200000	-0.94700000	-1.23400000	2.03800000	1.44600000	-1.15200000
C	0.50900000	-1.12800000	-0.41900000	0.52300000	1.62800000	-0.93500000
N	-0.22700000	-0.00100000	-0.25300000	-0.23400000	0.54800000	-1.24900000
C	-1.42600000	0.02900000	0.55500000	-1.67300000	0.53900000	-1.11300000
C	-2.68000000	0.10800000	-0.31300000	-2.11300000	-0.22700000	0.13300000
H	3.47400000	-2.81700000	-0.09300000	3.03900000	2.57000000	1.15400000
H	5.45100000	-1.74100000	1.50800000	4.14500000	0.75300000	2.91500000
O	0.20400000	-2.22200000	0.03400000	0.07400000	2.68500000	-0.51500000
C	-4.00400000	0.14100000	0.49500000	-3.65100000	-0.26500000	0.33800000
F	-2.65800000	1.23200000	-1.09100000	-1.68500000	-1.52500000	0.07500000
F	-2.75500000	-0.95700000	-1.16200000	-1.57100000	0.31700000	1.26000000
F	-4.02600000	1.20400000	1.31500000	-4.24800000	-0.85200000	-0.71100000
F	-4.12600000	-0.97000000	1.23900000	-4.13700000	0.98000000	0.45800000
F	-5.06100000	0.21900000	-0.32400000	-3.96900000	-0.95100000	1.44300000
H	1.74600000	-0.11500000	-1.92900000	2.47200000	2.43800000	-1.25200000

H	1.96900000	-1.86800000	-1.78800000	2.25400000	0.86600000	-2.04400000
H	0.16300000	0.87500000	-0.57500000	0.23400000	-0.28300000	-1.58900000
H	-1.41000000	0.89300000	1.22000000	-2.12900000	0.07200000	-1.98600000
H	-1.47200000	-0.88000000	1.15400000	-2.02700000	1.56600000	-1.03500000
